# Supplementary material for: Kinase inhibition profiles as a tool to identify kinases for specific phosphorylation sites
Source: Nat Commun. 2020 Apr 3;11:1684. doi: 10.1038/s41467-020-15428-0 (PMC7125195; doi:10.1038/s41467-020-15428-0)
Supplement: Supplementary file 1 — Supplementary Information [file 41467_2020_15428_MOESM1_ESM.pdf]

Supplementary Information

Kinase inhibition profiles as a tool to identify kinases for specific phosphorylation sites

Watson *et al*.

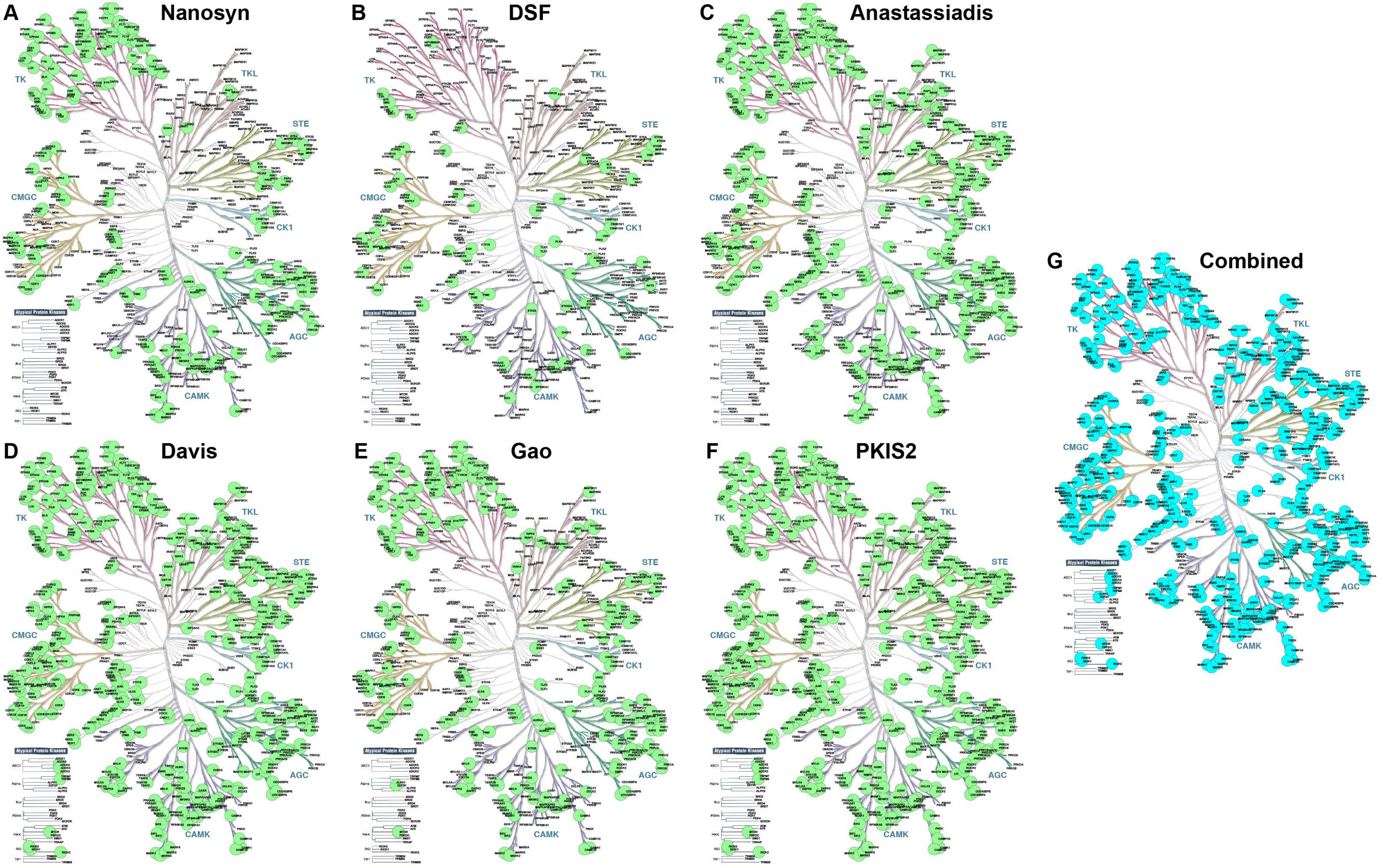

Supplementary Figure 1. Kinome trees showing all kinases included in the profiling datasets used in this work.

A. Nanosyn set. **B**. DSF set. **C**. Anastassiadis set. **D**. Davis set. **E**. Gao set. **F**. PKIS2 (Drewry) set. **G**. All sets combined. Circles indicate kinases included in the set. Kinases are named according to their HGNC gene names. Kinome trees were produced using KinMap[1], and the underlying tree illustration is reproduced courtesy of Cell Signaling Technology, Inc. (www.cellsignal.com).

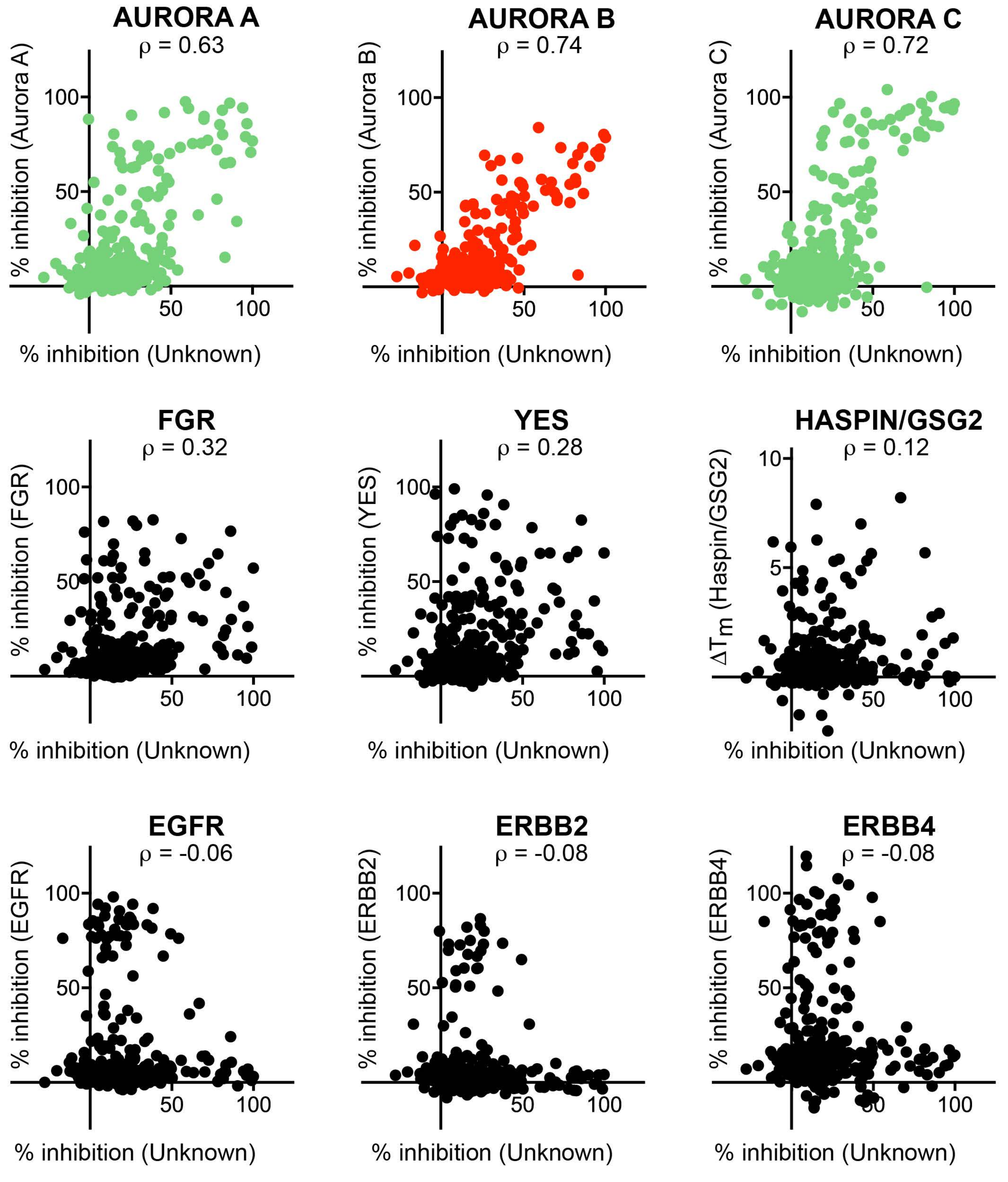

Supplementary Figure 2. Selected kinase inhibition correlation plots for the H3S28ph kinase screen using the PKIS1 inhibitor set.

KiPIK results obtained from the H3S28ph kinase screen were compared to profiling data from Elkins *et al.* (Nanosyn 1 μM for Aurora A, Aurora B, Aurora C, FGR, YES, EGFR, ERBB2 and ERBB4; DSF for Haspin/GSG2)[2]. Profiling data are on the y axis; KiPIK (unknown) data on the x axis. Pearson correlation coefficients (ρ) are shown. The best correlation screen-wide is shown in red (Aurora B), and closely related kinases in green.

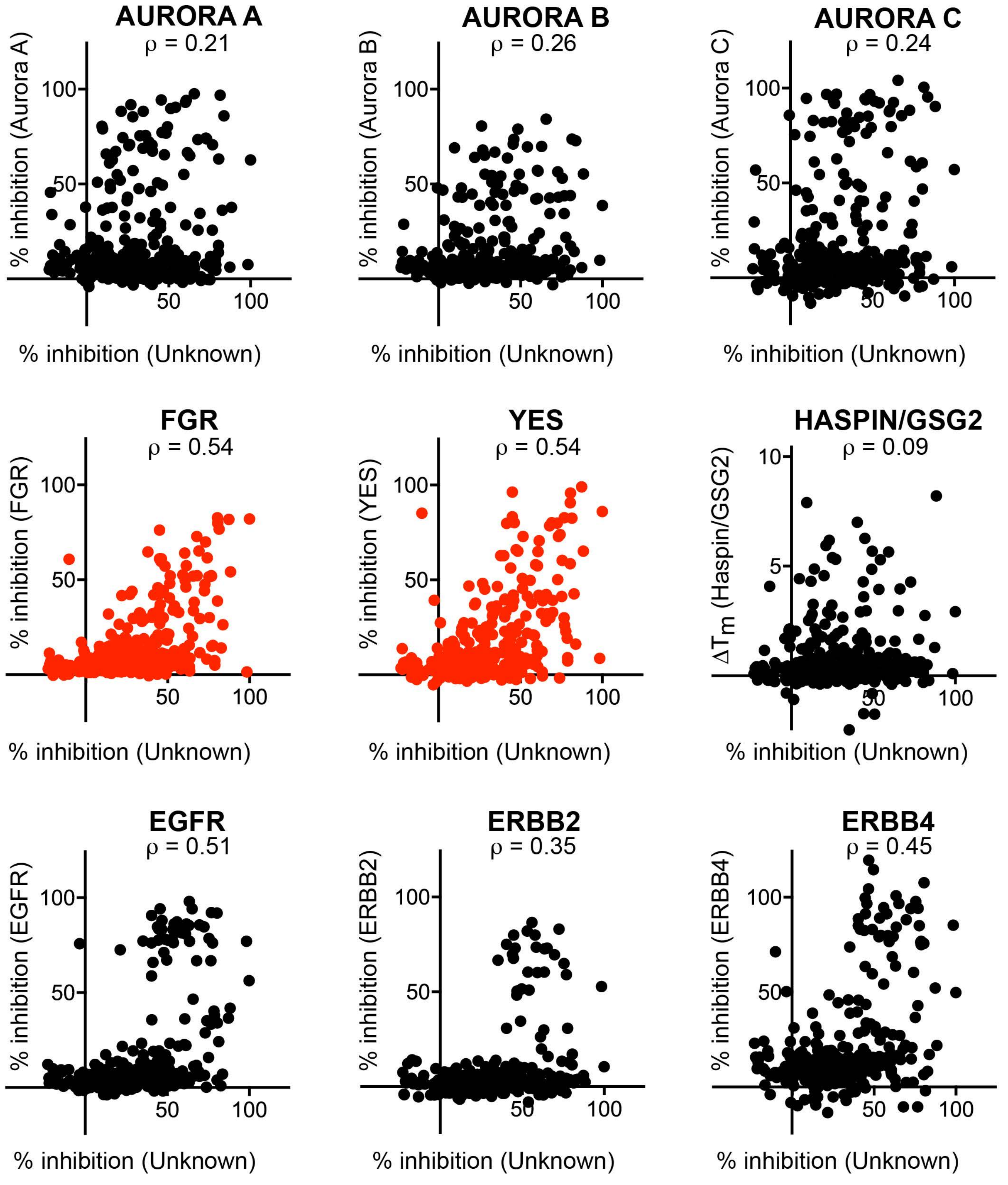

Supplementary Figure 3. Selected kinase inhibition correlation plots for the Integrin β1A Y795ph kinase screen using the PKIS1 inhibitor set.

KiPIK results obtained from the Integrin β1A Y795ph kinase screen were compared to profiling data from Elkins *et al.* (Nanosyn 1 µM for Aurora A, Aurora B, Aurora C, FGR, YES, EGFR, ERBB2 and ERBB4; DSF for Haspin/GSG2)[2]. Profiling data are on the y axis; KiPIK (unknown) data on the x axis. Pearson correlation coefficients (ρ) are shown. The best correlations screen-wide are shown in red (the Src family kinases FGR and YES).

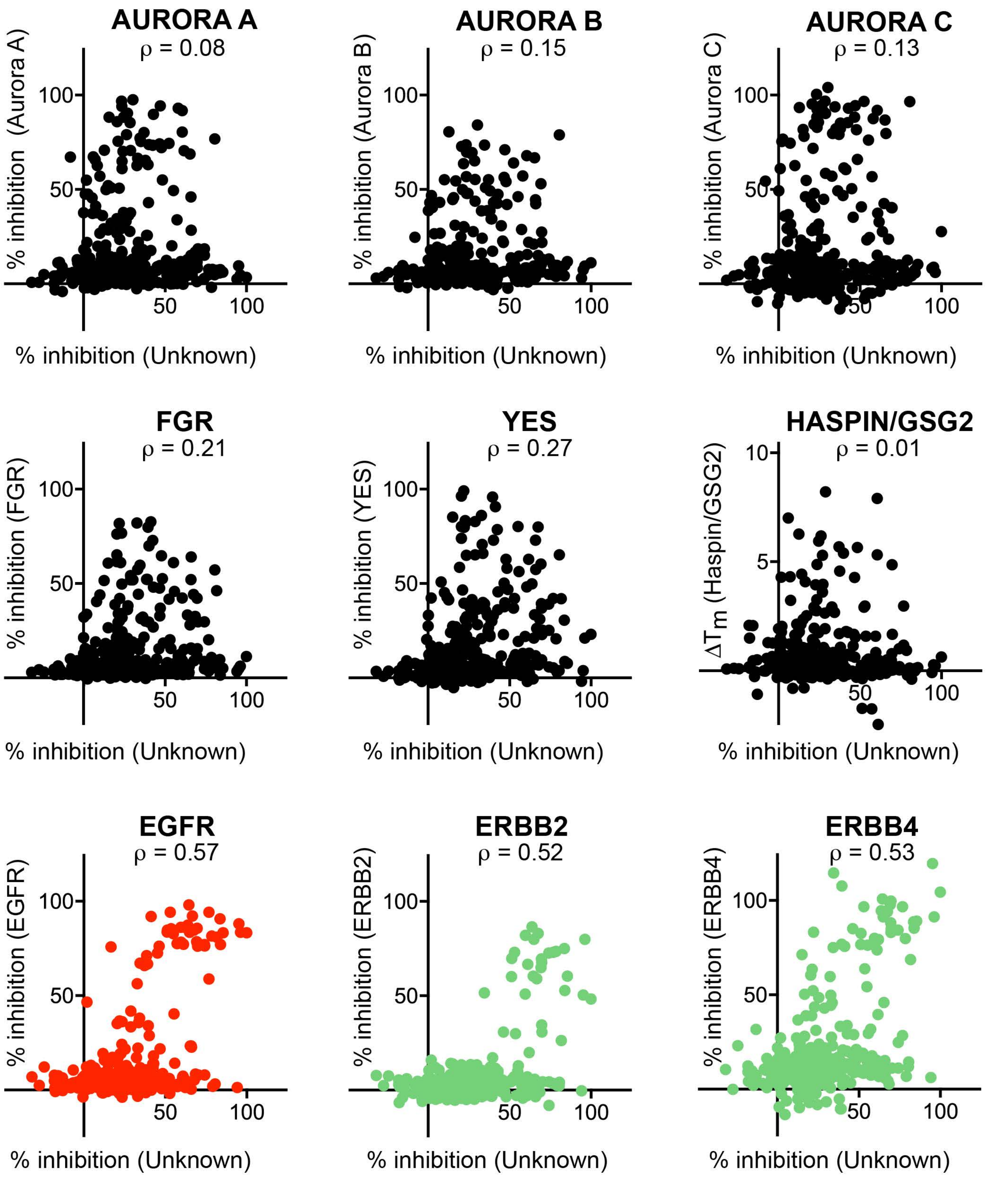

Supplementary Figure 4. Selected kinase inhibition correlation plots for the EGFR Y1016ph kinase screen using the PKIS1 inhibitor set.

KiPIK results obtained from the EGFR Y1016ph kinase screen were compared to profiling data from Elkins *et al.* (Nanosyn 1 µM for Aurora A, Aurora B, Aurora C, FGR, YES, EGFR, ERBB2 and ERBB4; DSF for Haspin/GSG2)[2]. Profiling data are on the y axis; KiPIK (unknown) data on the x axis. Pearson correlation coefficients (ρ) are shown. The best correlation screen-wide is shown in red (EGFR) and related kinases (ERBB2 and ERRB4) are shown in green.

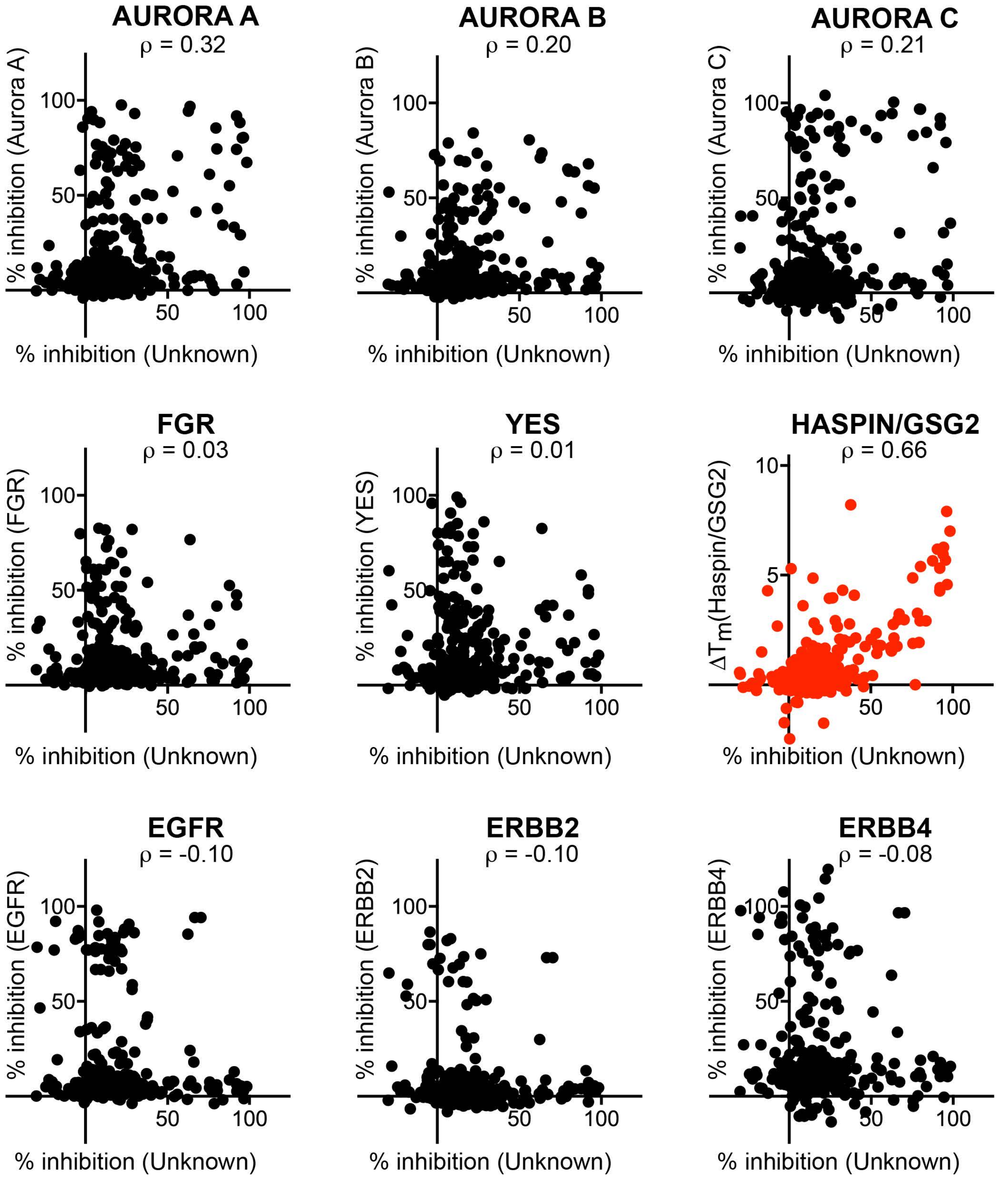

Supplementary Figure 5. Selected kinase inhibition correlation plots for the H3T3ph kinase screen using the PKIS1 inhibitor set.

KiPIK results obtained from the H3T3ph kinase screen were compared to profiling data from Elkins *et al.* (Nanosyn 1 μM for Aurora A, Aurora B, Aurora C, FGR, YES, EGFR, ERBB2 and ERBB4; DSF for Haspin/GSG2)[2]. Profiling data are on the y axis; KiPIK (unknown) data on the x axis. Pearson correlation coefficients (ρ) are shown. The best correlation screen-wide is shown in red (Haspin/GSG2).

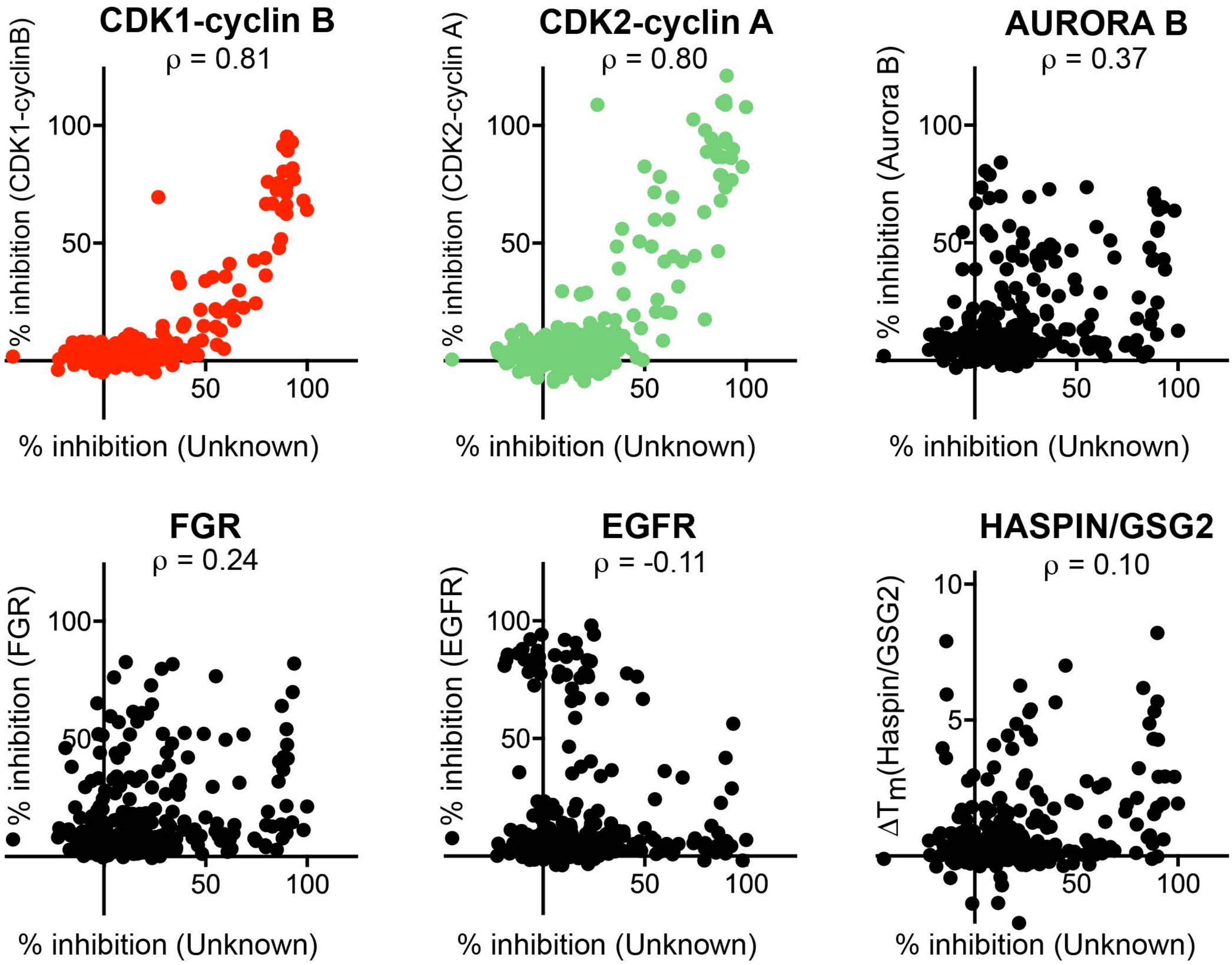

Supplementary Figure 6. Selected kinase inhibition correlation plots for the INCENP S446ph kinase screen using the PKIS1 inhibitor set.

KiPIK results obtained from the INCENP S446ph kinase screen were compared to profiling data from Elkins *et al.* (Nanosyn 1 µM for Aurora A, Aurora B, Aurora C, FGR, YES, EGFR, ERBB2 and ERBB4; DSF for Haspin/GSG2)[2]. Profiling data are on the y axis; KiPIK (unknown) data on the x axis. Pearson correlation coefficients (ρ) are shown. The best correlation screen-wide is shown in red (CDK1-cyclin B) and the closest related kinase (CDK2-cyclin A) in green.

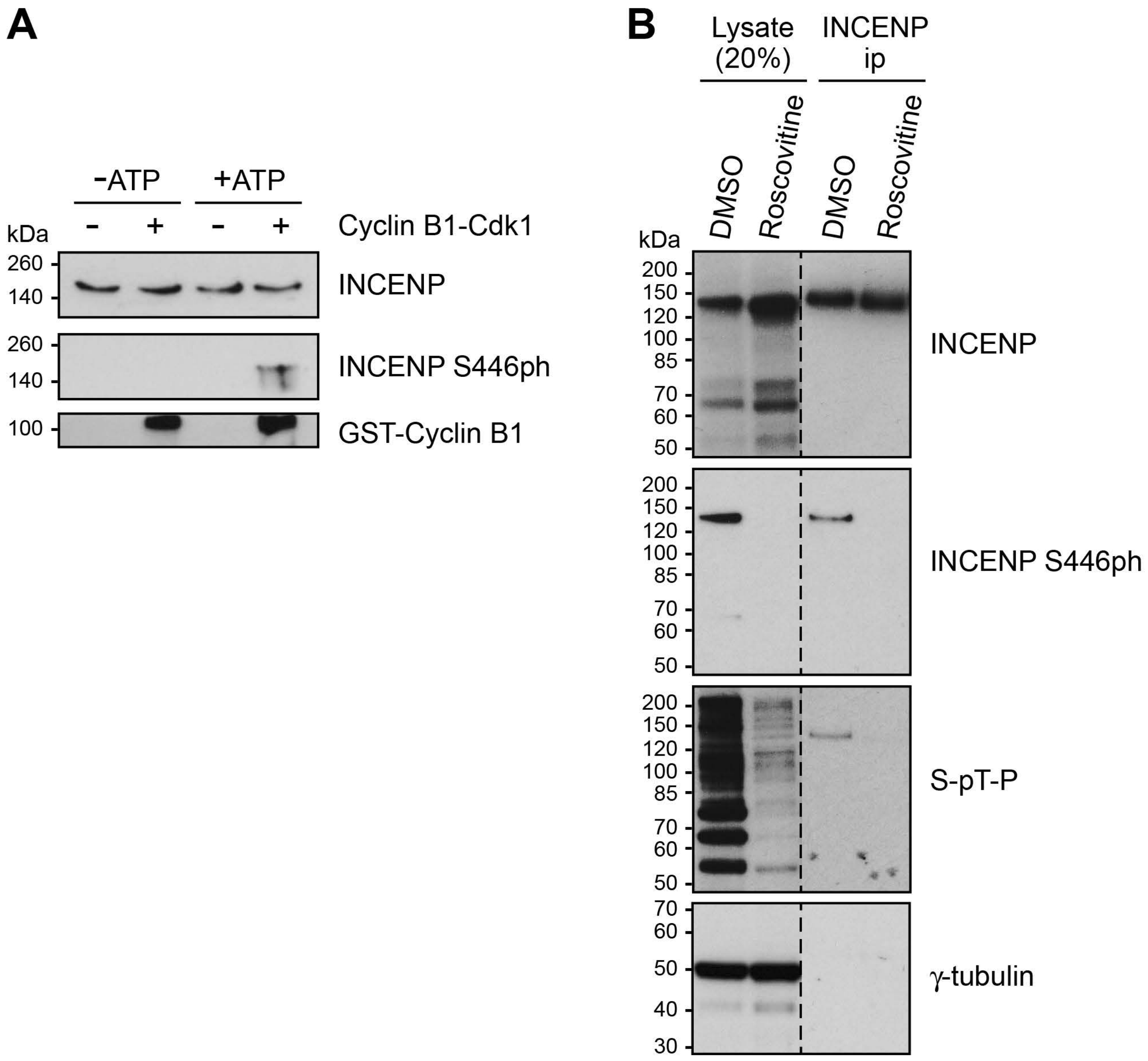

Supplementary Figure 7. INCENP S446 phosphorylation *in vitro* and in cells.

A. Recombinant full length human INCENP-6His (purified as a complex with human Aurora B) is phosphorylated by purified Cyclin B1-Cdk1 *in vitro*. Reactions were carried out in the presence of 0.5 µM ZM447439 to inhibit Aurora B kinase activity. After SDS-PAGE, immunoblotting with antibodies to INCENP, INCENP S446ph and Cyclin B1 was carried out. Parallel blots of the same samples were probed for INCENP and INCENP S446ph. Note that we used INCENP-Aurora B complexes because the solubility of recombinant full-length INCENP is reported to depend on the coexpression of Aurora B[3]. The experiment was done three times with similar results.

B. INCENP S446 phosphorylation in cells is prevented by the Cdk inhibitor Roscovitine. INCENP was immunoprecipitated from nocodazole-arrested mitotic HeLa 1C8 cell lysates[4] that were treated with or without Roscovitine for 2 h. After SDS-PAGE, immunoblotting with antibodies to INCENP, INCENP S446ph, the S-pT-P motif (which detects a subset of Cdk1 phosphorylation sites), or γ-tubulin was carried out. The experiment was performed once.
Source data are provided as a Source Data file.

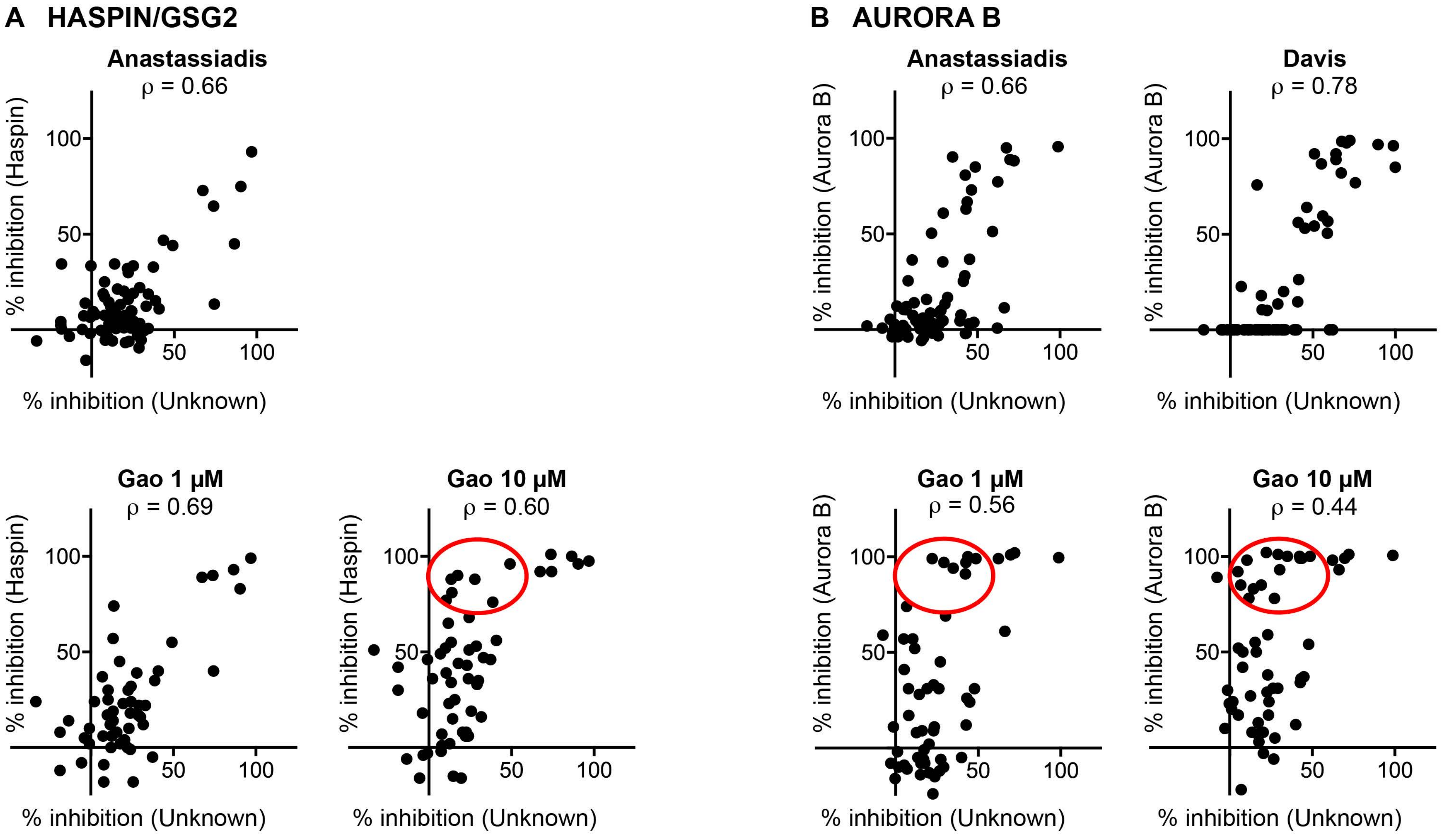

Supplementary Figure 8. Examples of kinase inhibition correlation plots for Haspin/GSG2 and Aurora B.

KiPIK results obtained from the (**A**) H3T3ph kinase screen and (**B**) H3S28ph kinase screen, compared to profiling data from Anastassiadis *et al.*[5], Davis *et al.*[6], and Gao *et al.*[7]. Profiling data are on the y axis; KiPIK (unknown) data on the x axis. Pearson correlation coefficients (ρ) are shown.

Red ellipses indicate compounds that give particulary high %inhibition values in the profiling data compared to the KiPIK data due to the use of relatively high compound concentration in the Gao profiling experiment.

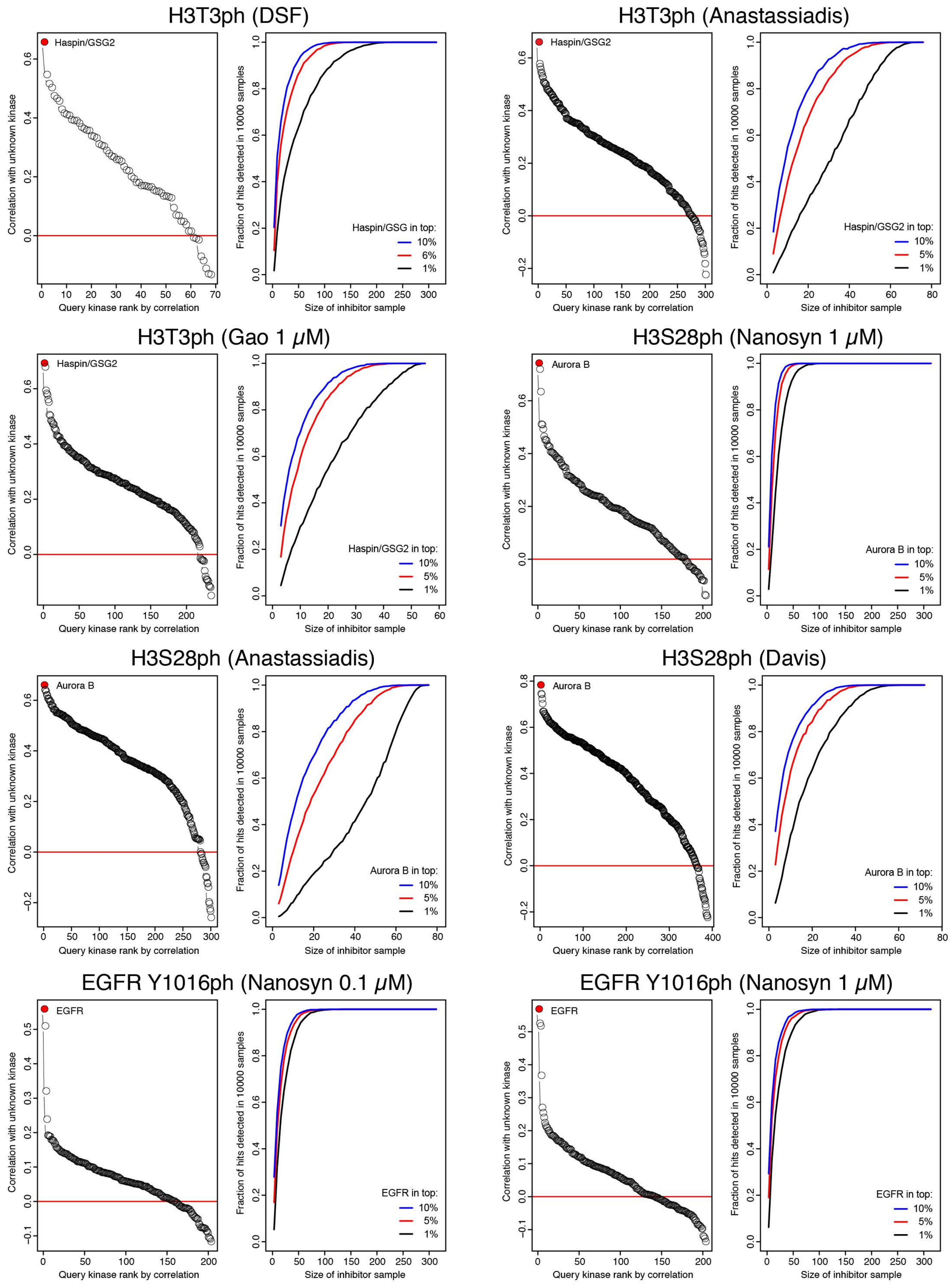

Supplementary Figure 9. Downsampling reveals the effect of inhibitor library size on the robustness of KiPIK.

The righthand plot of each pair shows the fraction of times that the expected kinase was found to be in the top 1%, 5% or 10% of all kinases in the profiling dataset, as a function of inhibitor library size. This allows results from different datasets (which have different numbers of kinases) to be compared. Lefthand plots show the ranked correlation scores for the unknown kinase with each query kinase in the library.

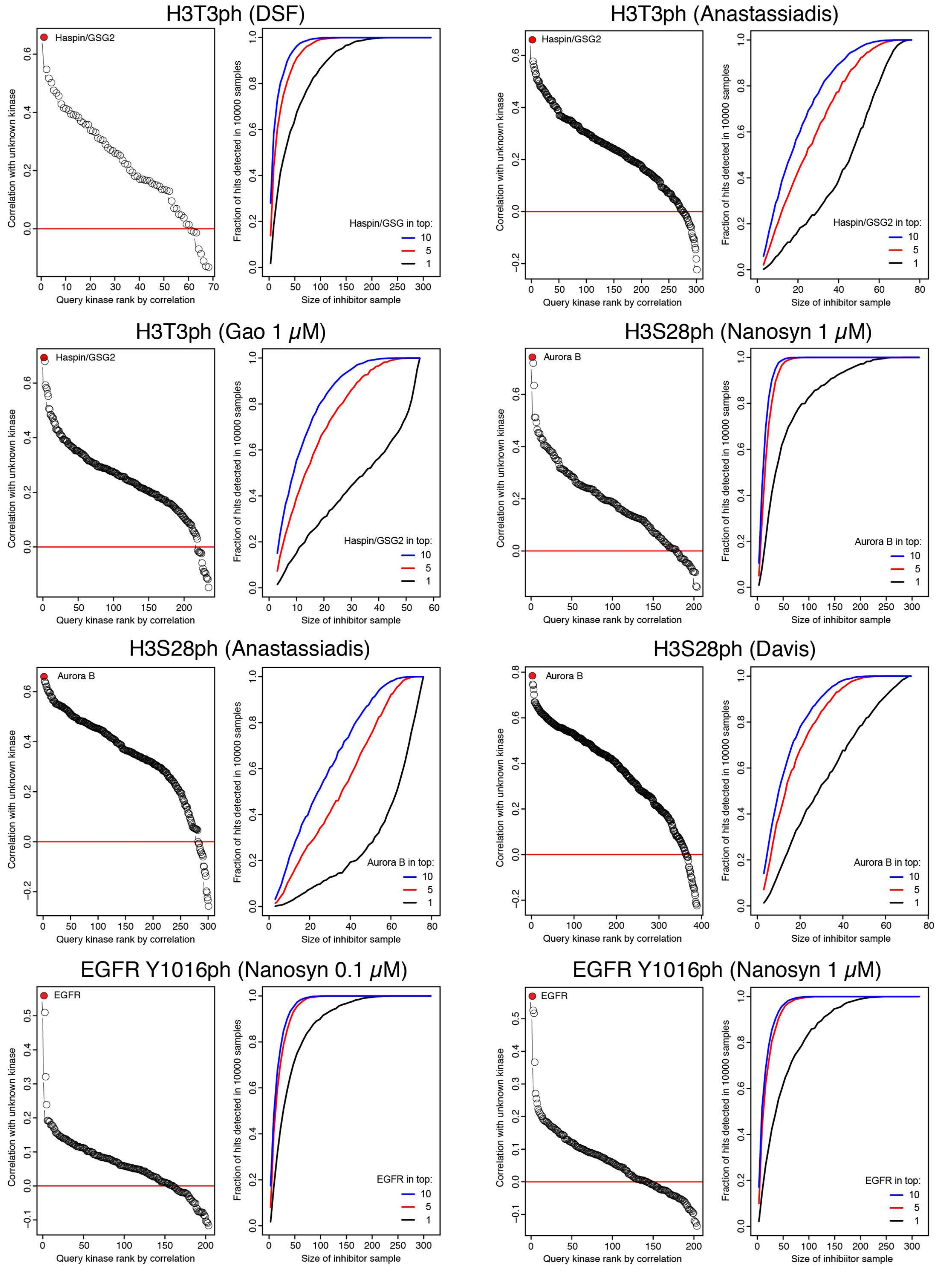

Supplementary Figure 10. Downsampling reveals the effect of inhibitor library size on the robustness of KiPIK.

The righthand plot of each pair shows the fraction of times that the expected kinase was found to be ranked in the top 1, 5 or 10 of all kinases in the profiling dataset, as a function of inhibitor library size. This does not take into account of the fact that different datasets have different numbers of kinases. Lefthand plots show the ranked correlation scores for the expected kinase with each query kinase in the library.

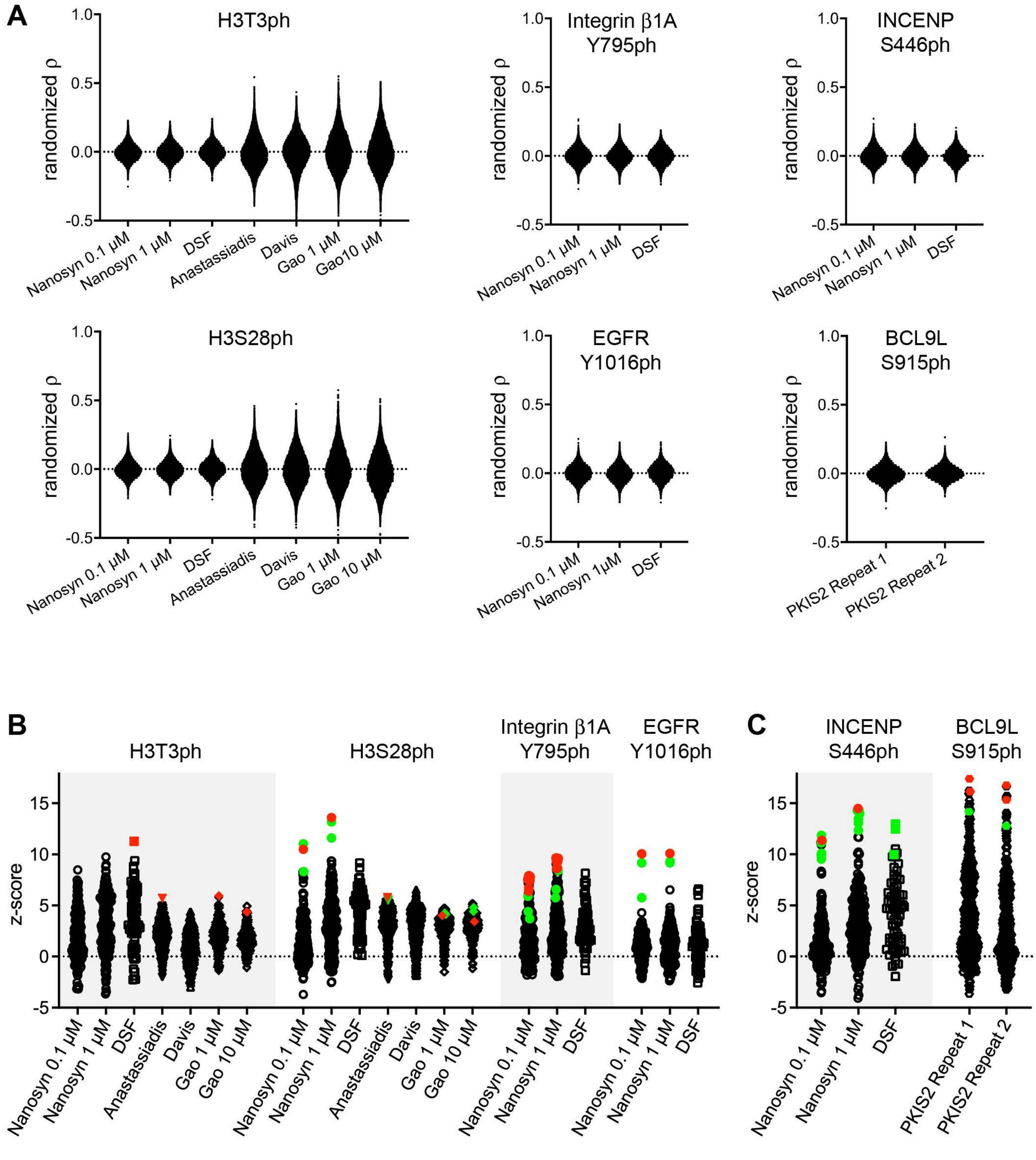

Supplementary Figure 11. Calculation of *z*-scores for KiPIK screens.

A. Null distributions of Pearson correlation coefficients (ρ) for each KiPIK screen calculated by random permutation of inhibitor labels (see Methods for details).

B. *Z*-scores for all kinases in all profiling datasets in KiPIK screens for known kinases. Expected hit kinases are shown in red (Haspin/GSG2 for H3T3ph; Aurora B for H3S28ph; Src-family kinases for Integrin β1A Y795ph; and EGFR for EGFR Y1016ph). Related kinases are shown in green (Aurora A/C for H3S28ph; FRK, BRK and SRMS for Integrin β1A Y795ph; and ERBB2/4 for EGFR Y1016ph).

C. *Z*-scores for all kinases in profiling datasets in KiPIK screens for unknown kinases. Overall top hit kinases are shown in red (Cdk1-Cyclin B for INCENP S446ph; PKA-α/β for BCL9L S915ph). Related kinases are shown in green (other Cdk-family kinases for INCENP S446ph; PRKX for BCL9L S915ph).

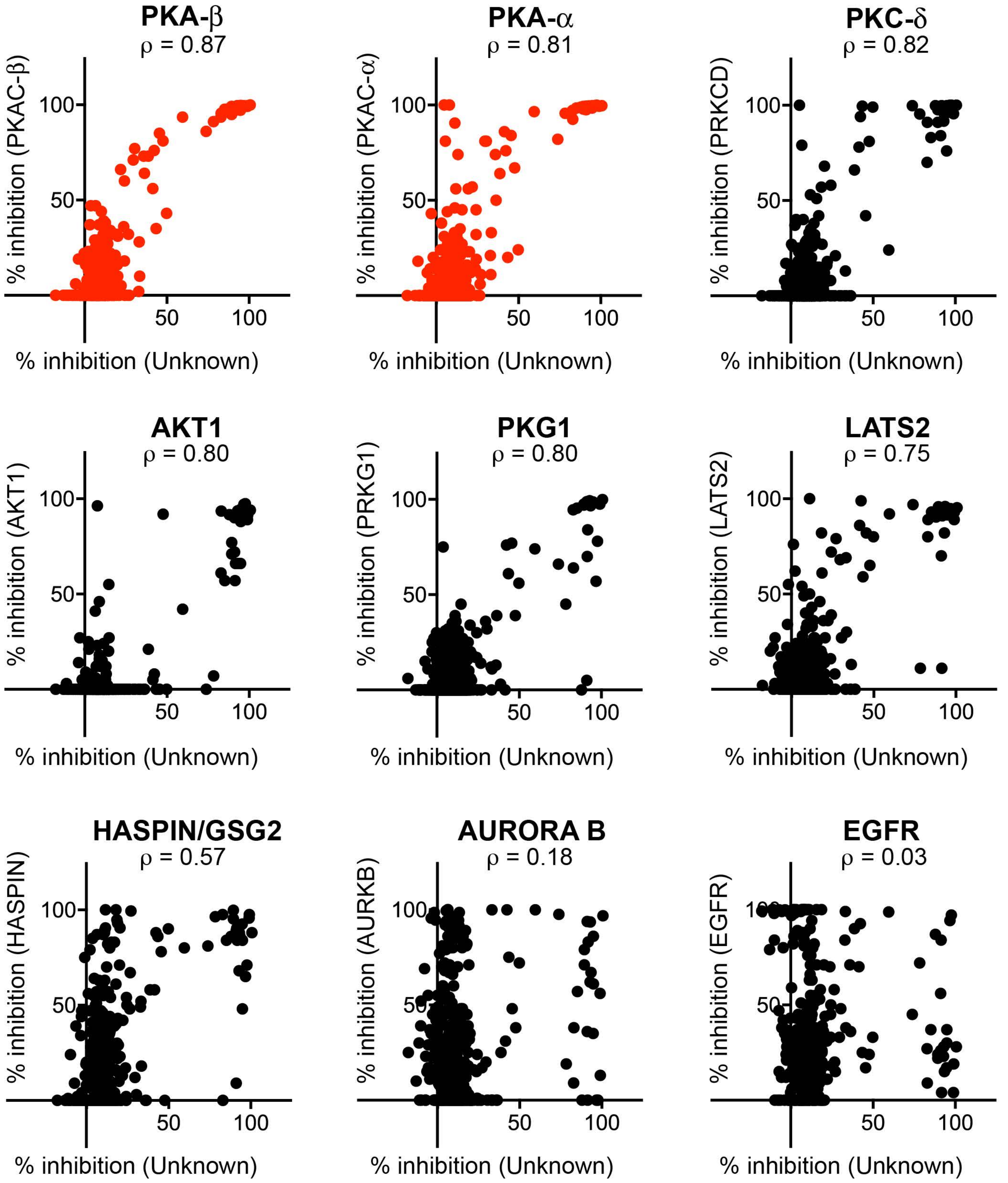

Supplementary Figure 12. Selected kinase inhibition correlation plots for the BCL9L S915 kinase screen using the PKIS2 inhibitor set.

KiPIK results obtained from the BCL9L S915ph kinase screen repeat 1 were compared to profiling data from Drewery *et al.*[8]. Profiling data are on the y axis; KiPIK (unknown) data on the x axis. Pearson correlation coefficients (ρ) are shown. The best correlations screen-wide are shown in red (PKA-α/β). Source data are provided as a Source Data file.

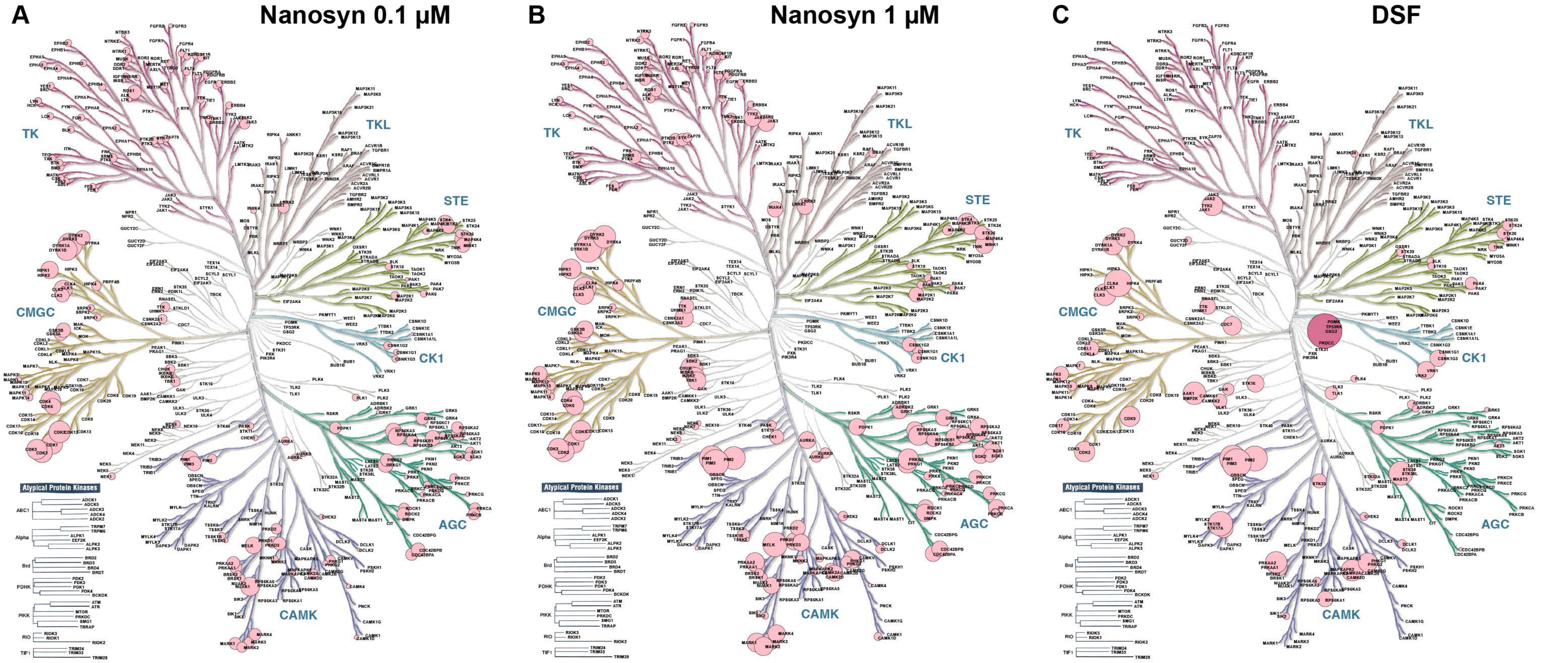

Supplementary Figure 13. Kinome trees showing KiPIK correlation coefficients for the H3T3ph screen with the PKIS1 inhibitor library.

A. Nanosyn screen at 0.1 µM inhibitor concentration. **B**. Nanosyn screen at 1 µM inhibitor concentration. **C**. DSF screen. Circle sizes indicate correlation coefficients (from no circle at $\rho = 0$ to the largest circle at $\rho = 1.0$). The highest scoring kinase overall, Haspin/GSG2, is indicated by the dark pink circle. Kinases are named according to their HGNC gene names. Kinome trees were produced using KinMap[1], and the underlying tree illustration is reproduced courtesy of Cell Signaling Technology, Inc. (www.cellsignal.com). Source data are provided as a Source Data file.

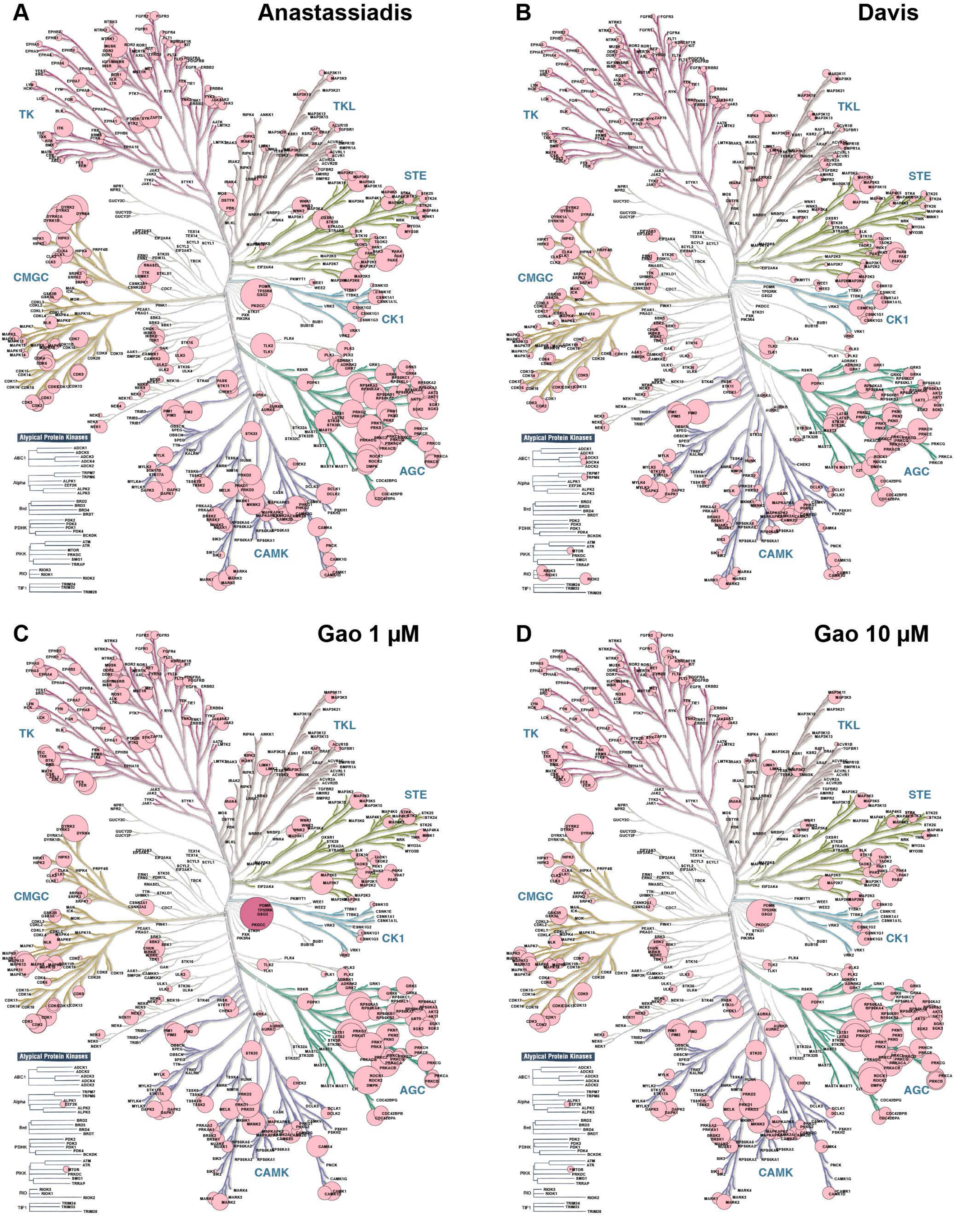

Supplementary Figure 14. Kinome trees showing KiPIK correlation coefficients for the H3T3ph screen with the custom inhibitor library.

A. Anastassiadis set. **B**. Davis set. **C**. Gao set at 1 µM inhibitor concentration. **D**. Gao set at 10 µM inhibitor concentration. Circle sizes indicate KiPIK correlation coefficients (from no circle at $\rho = 0$ to the largest circle at $\rho = 1.0$). The highest scoring kinase, Haspin/GSG2, is shown as a dark pink circle. Kinases are named according to their HGNC gene names. Kinome trees were produced using KinMap[1], and the underlying tree illustration is reproduced courtesy of Cell Signaling Technology, Inc. (www.cellsignal.com). Source data are provided as a Source Data file.

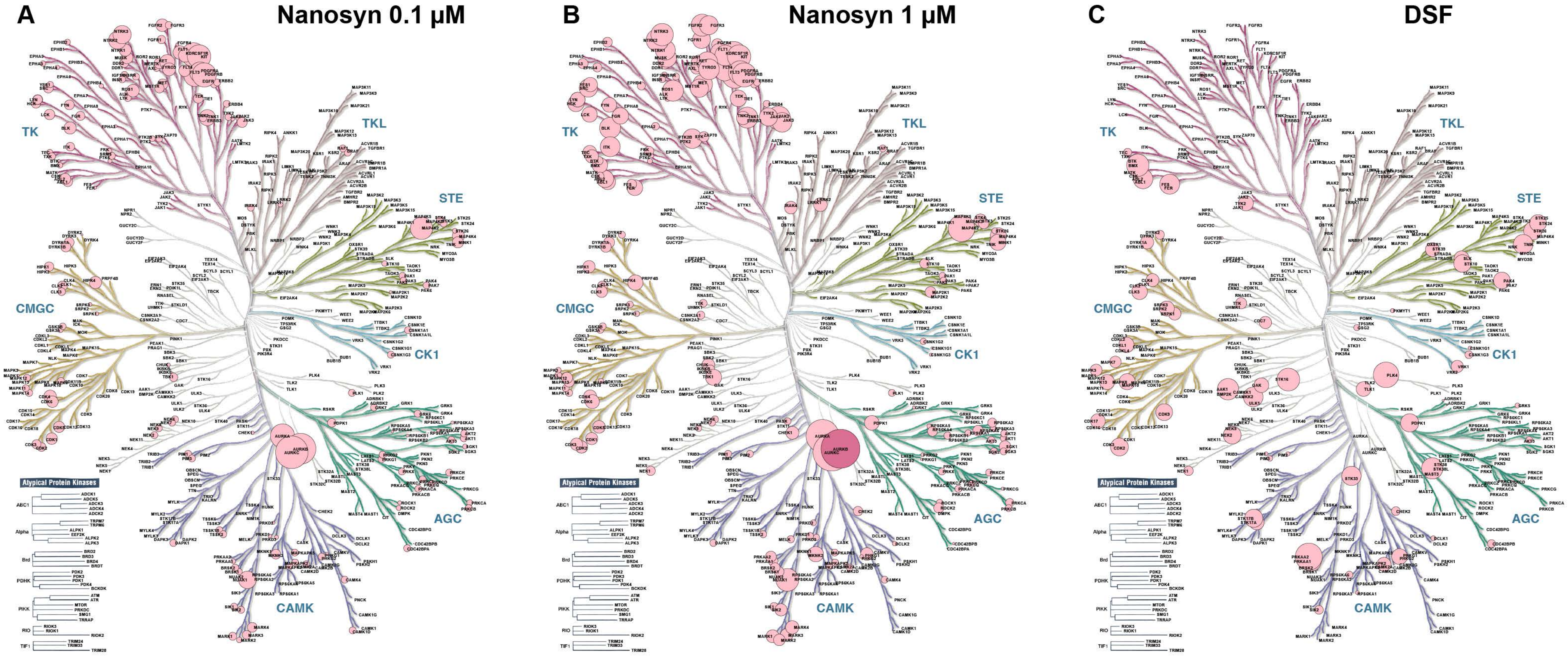

Supplementary Figure 15. Kinome trees showing KiPIK correlation coefficients for the H3S28ph screen with the PKIS1 inhibitor library.

A. Nanosyn screen at 0.1 μM inhibitor concentration. **B**. Nanosyn screen at 1 μM inhibitor concentration. **C**. DSF screen. Circle sizes indicate KiPIK correlation coefficients (from no circle at $\rho = 0$ to the largest circle at $\rho = 1.0$). The highest scoring kinase overall, Aurora B/AURKB, is indicated by the dark pink circle. Kinases are named according to their HGNC gene names. Kinome trees were produced using KinMap[1], and the underlying tree illustration is reproduced courtesy of Cell Signaling Technology, Inc. (www.cell signal.com). Source data are provided as a Source Data file.

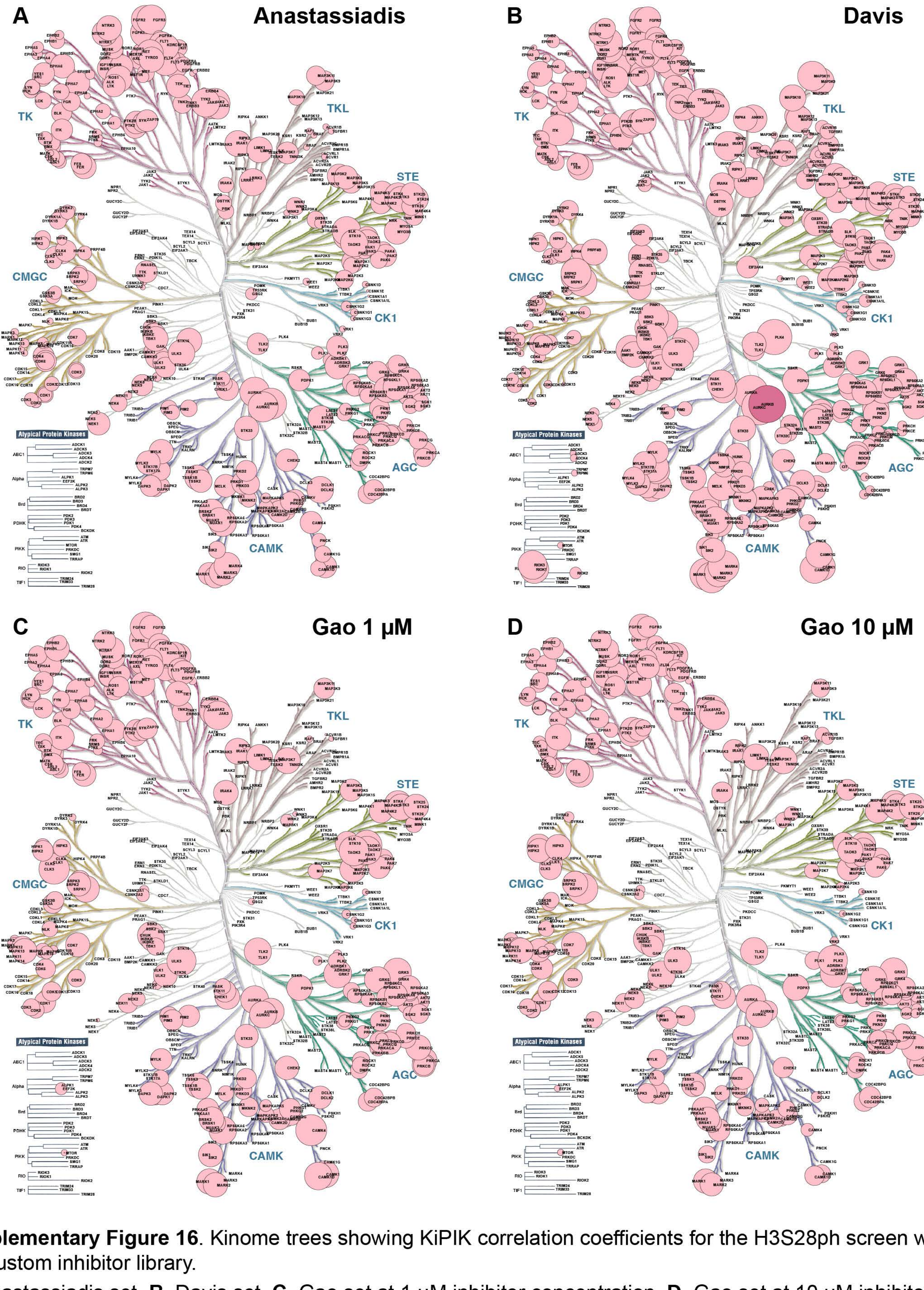

Supplementary Figure 16. Kinome trees showing KiPIK correlation coefficients for the H3S28ph screen with the custom inhibitor library.

A. Anastassiadis set. **B**. Davis set. **C**. Gao set at 1 µM inhibitor concentration. **D**. Gao set at 10 µM inhibitor concentration. Circle sizes indicate KiPIK correlation coefficients (from no circle at ρ = 0 to the largest circle at ρ = 1.0). The highest scoring kinase overall, Aurora B/AURKB, is shown as a dark pink circle. Kinases are named according to their HGNC gene names. Kinome trees were produced using KinMap[1], and the underlying tree illustration is reproduced courtesy of Cell Signaling Technology, Inc. (www.cellsignal.com). Source data are provided as a Source Data file.

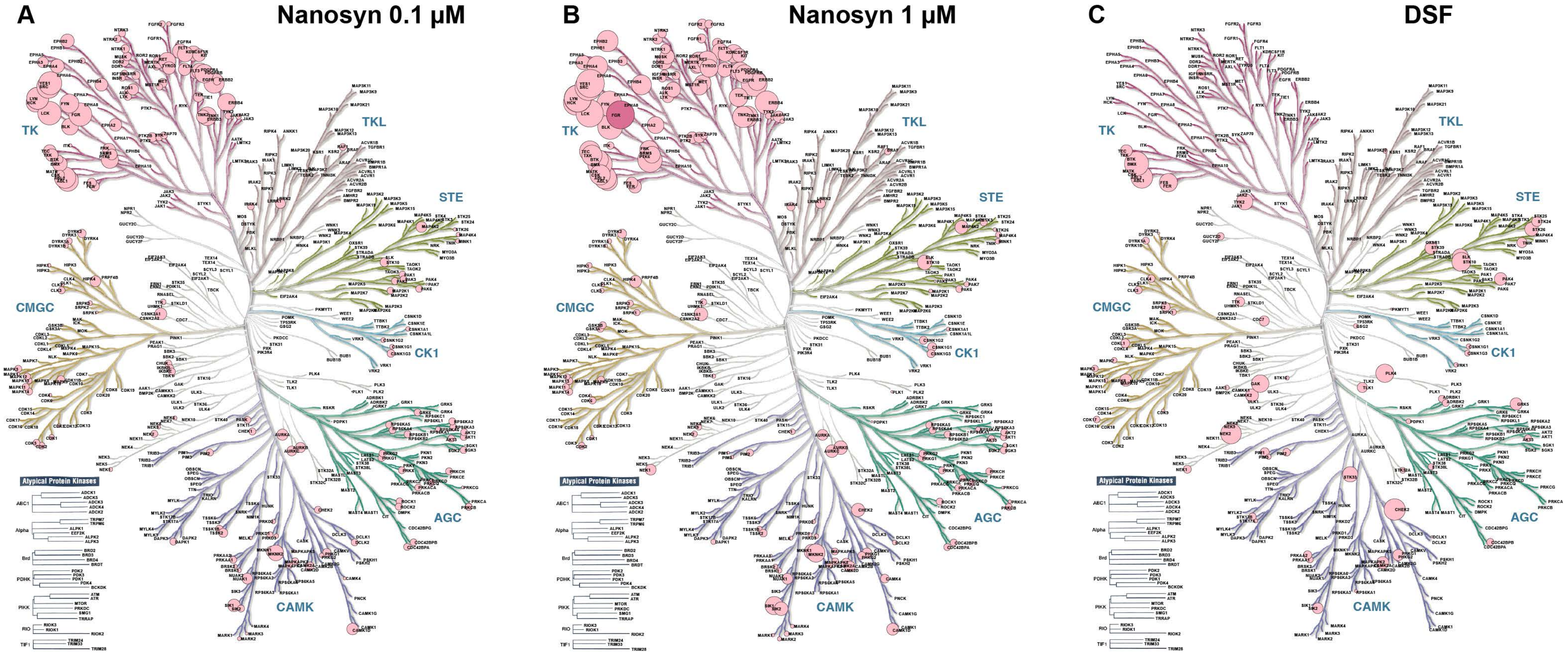

Supplementary Figure 17. Kinome trees showing KiPIK correlation coefficients for the Integrin β1A Y795ph screen with the PKIS1 inhibitor library.

A. Nanosyn screen at 0.1 µM inhibitor concentration. **B**. Nanosyn screen at 1 µM inhibitor concentration. **C**. DSF screen. Circle sizes indicate KiPIK correlation coefficients (from no circle at $\rho = 0$ to the largest circle at $\rho = 1.0$). The highest scoring kinase overall, the SRC family kinase FGR, is indicated by the dark pink circle. Kinases are named according to their HGNC gene names. Kinome trees were produced using KinMap[1], and the underlying tree illustration is reproduced courtesy of Cell Signaling Technology, Inc. (www.cellsignal.com). Source data are provided as a Source Data file.

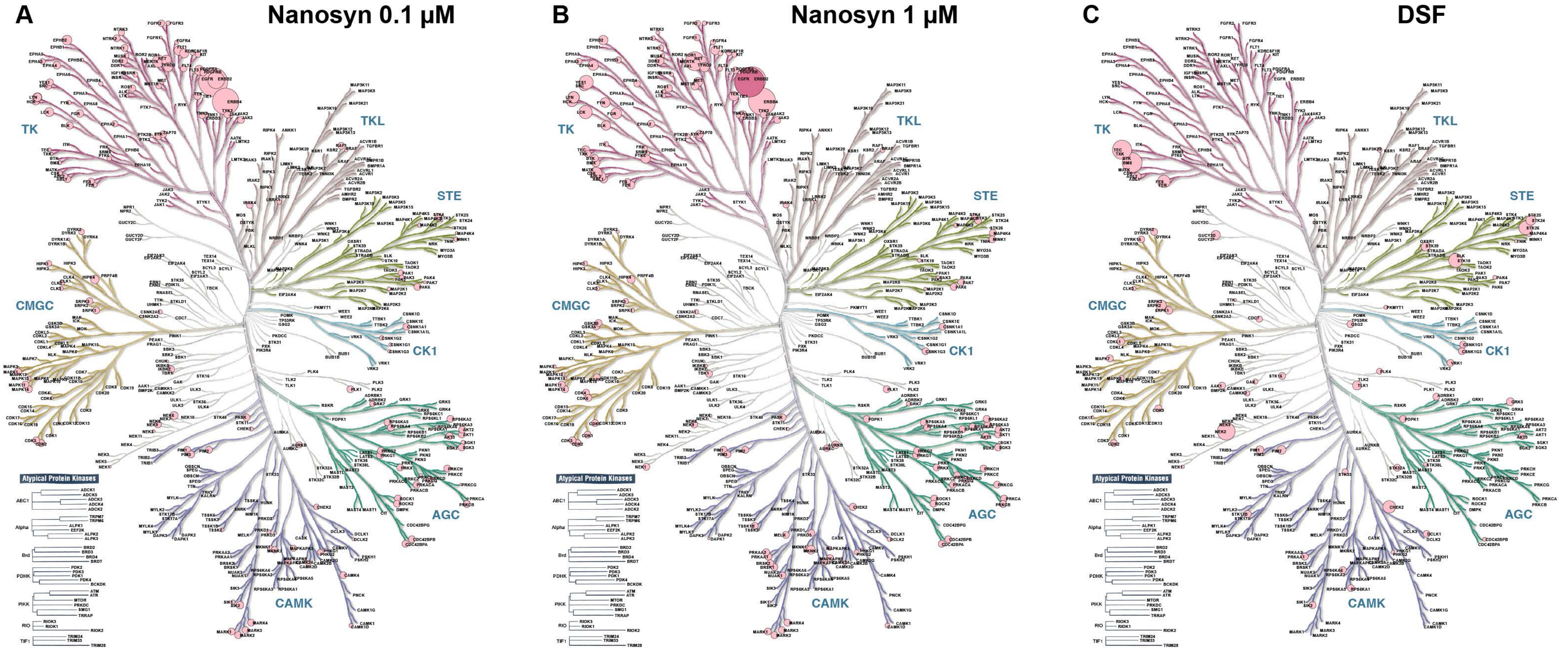

Supplementary Figure 18. Kinome trees showing KiPIK correlation coefficients for the EGFR Y1016ph screen with the PKIS1 inhibitor library.

A. Nanosyn screen at 0.1 µM inhibitor concentration. **B**. Nanosyn screen at 1 µM inhibitor concentration. **C**. DSF screen. Circle sizes indicate KiPIK correlation coefficients (from no circle at $\rho = 0$ to the largest circle at $\rho = 1.0$). The highest scoring kinase overall, EGFR, is indicated by the dark pink circle. Kinases are named according to their HGNC gene names. Kinome trees were produced using KinMap[1], and the underlying tree illustration is reproduced courtesy of Cell Signaling Technology, Inc. (www.cellsignal.com). Source data are provided as a Source Data file.

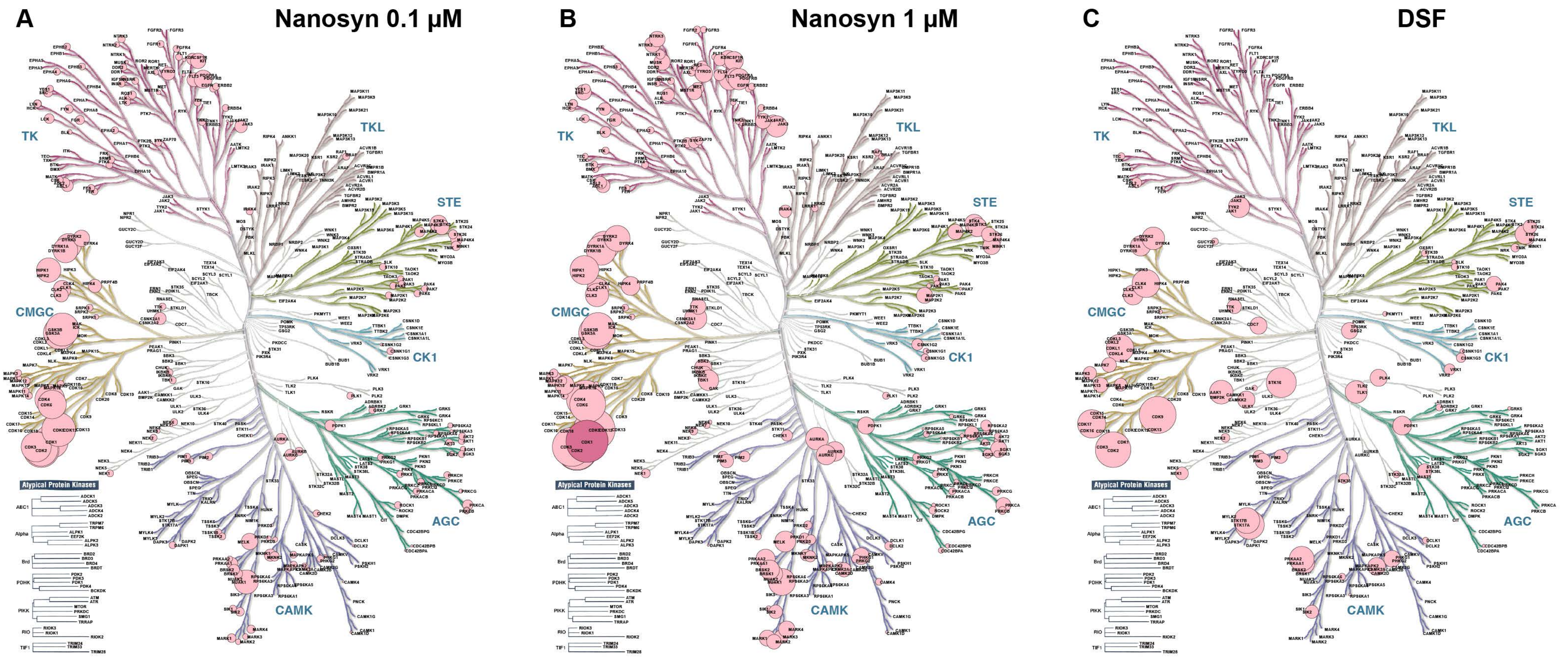

Supplementary Figure 19. Kinome trees showing KiPIK correlation coefficients for the INCENP S446ph screen with the PKIS1 inhibitor library.

A. Nanosyn screen at 0.1 µM inhibitor concentration. **B**. Nanosyn screen at 1 µM inhibitor concentration. **C**. DSF screen. Circle sizes indicate KiPIK correlation coefficients (from no circle at $\rho = 0$ to the largest circle at $\rho = 1.0$). The highest scoring kinase overall, CDK1-Cyclin B, is indicated by the dark pink circle. Kinases are named according to their HGNC gene names. Kinome trees were produced using KinMap[1], and the underlying tree illustration is reproduced courtesy of Cell Signaling Technology, Inc. (www.cellsignal.com). Source data are provided as a Source Data file.

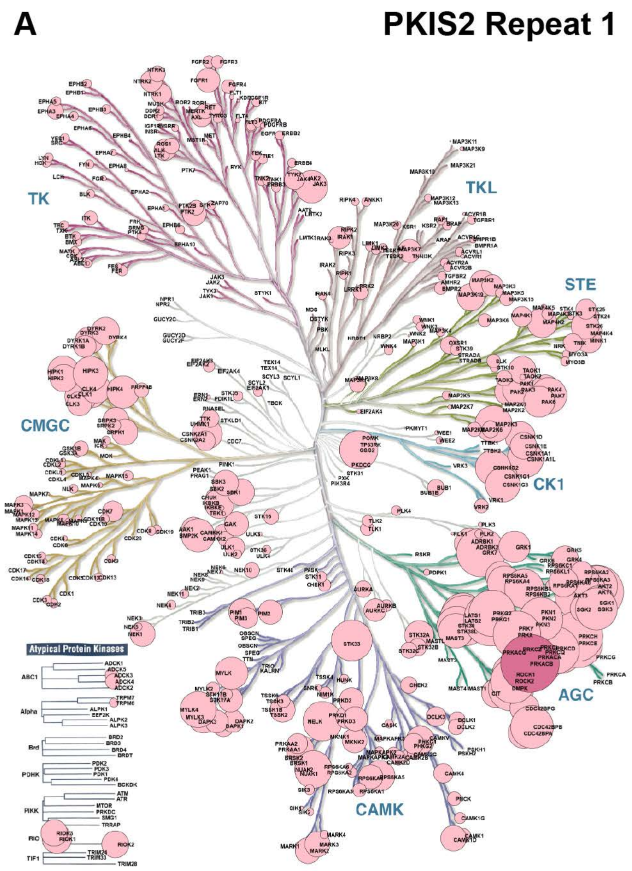

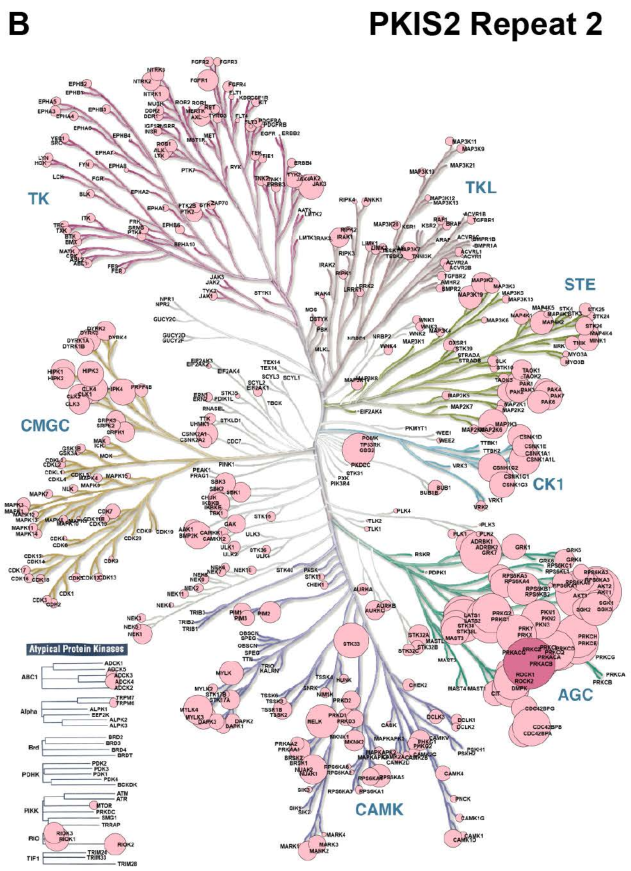

Supplementary Figure 20. Kinome trees showing KiPIK correlation coefficients for the BCL9L S915ph screen with the PKIS2 inhibitor library.

A. Repeat 1 with 399 inhibitors. **B**. Repeat 2 with 485 inhibitors. Circle sizes indicate KiPIK correlation coefficients (from no circle at ρ = 0 to the largest circle at ρ = 1.0). The highest scoring kinase overall, PKA-β, is indicated by the dark pink circle. Kinases are named according to their HGNC gene names. Kinome trees were produced using KinMap[1], and the underlying tree illustration is reproduced courtesy of Cell Signaling Technology, Inc. (www.cellsignal.com). Source data are provided as a Source Data file.

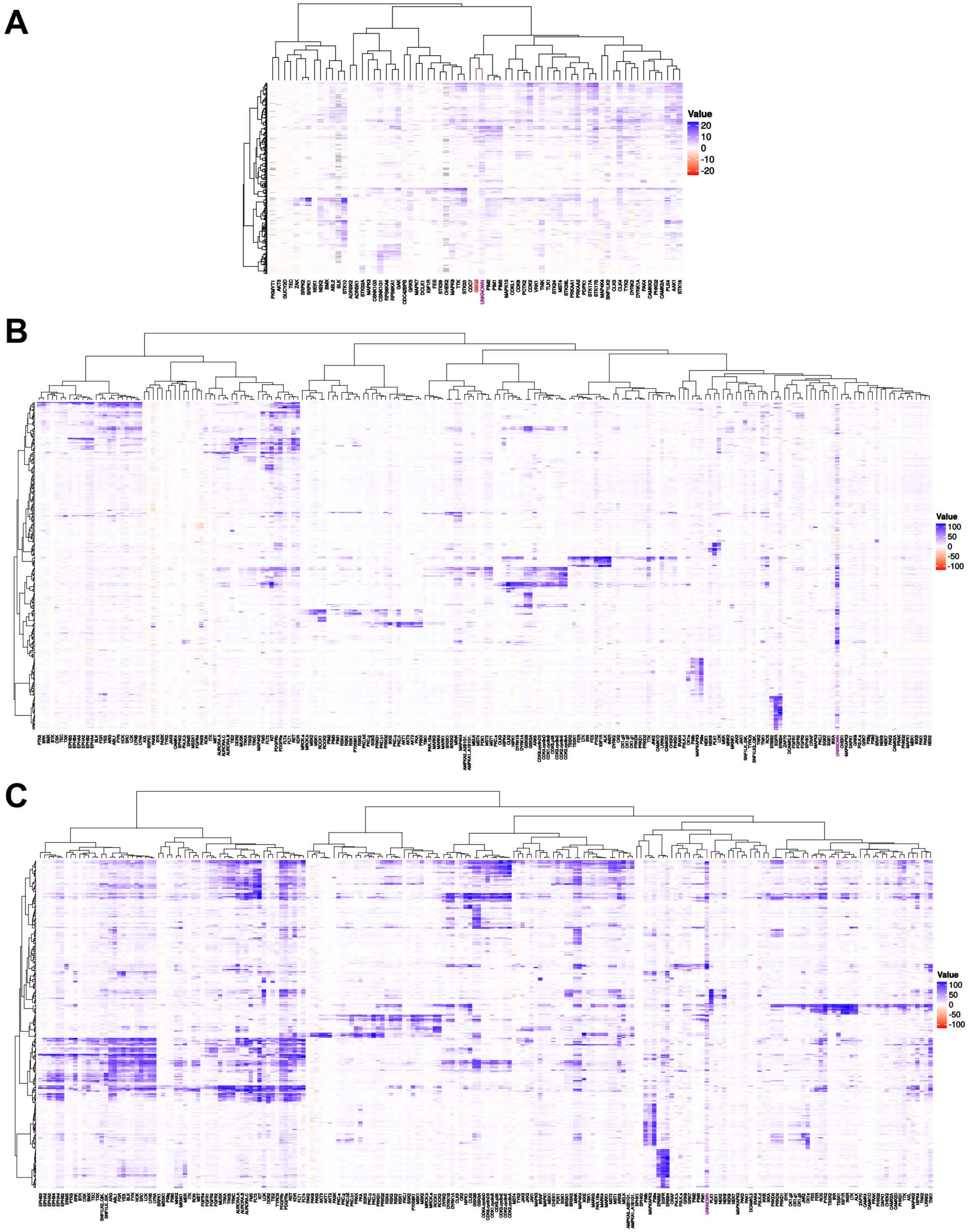

Supplementary Figure 21. Kinase dendrograms constructed from inhibition profiling data and KiPIK results for the H3T3ph kinase.

Pearson correlation coefficients calculated from pairwise comparisons of inhibition fingerprints were used to produce dendrograms for kinases (top) and kinase inhibitors (left side). Heat maps illustrate the percent inhibition score for each kinase-inhibitor pair (blue to red scale; grey indicates missing data). **A**. DSF dataset. Note that, for the DSF tree only, the KiPIK data are shown as %inhibition/10 so that they are on a similar scale. **B**. Nanosyn 0.1 μM dataset. **C**. Nanosyn 1 μM dataset. The “UNKNOWN” kinase is shown in purple.

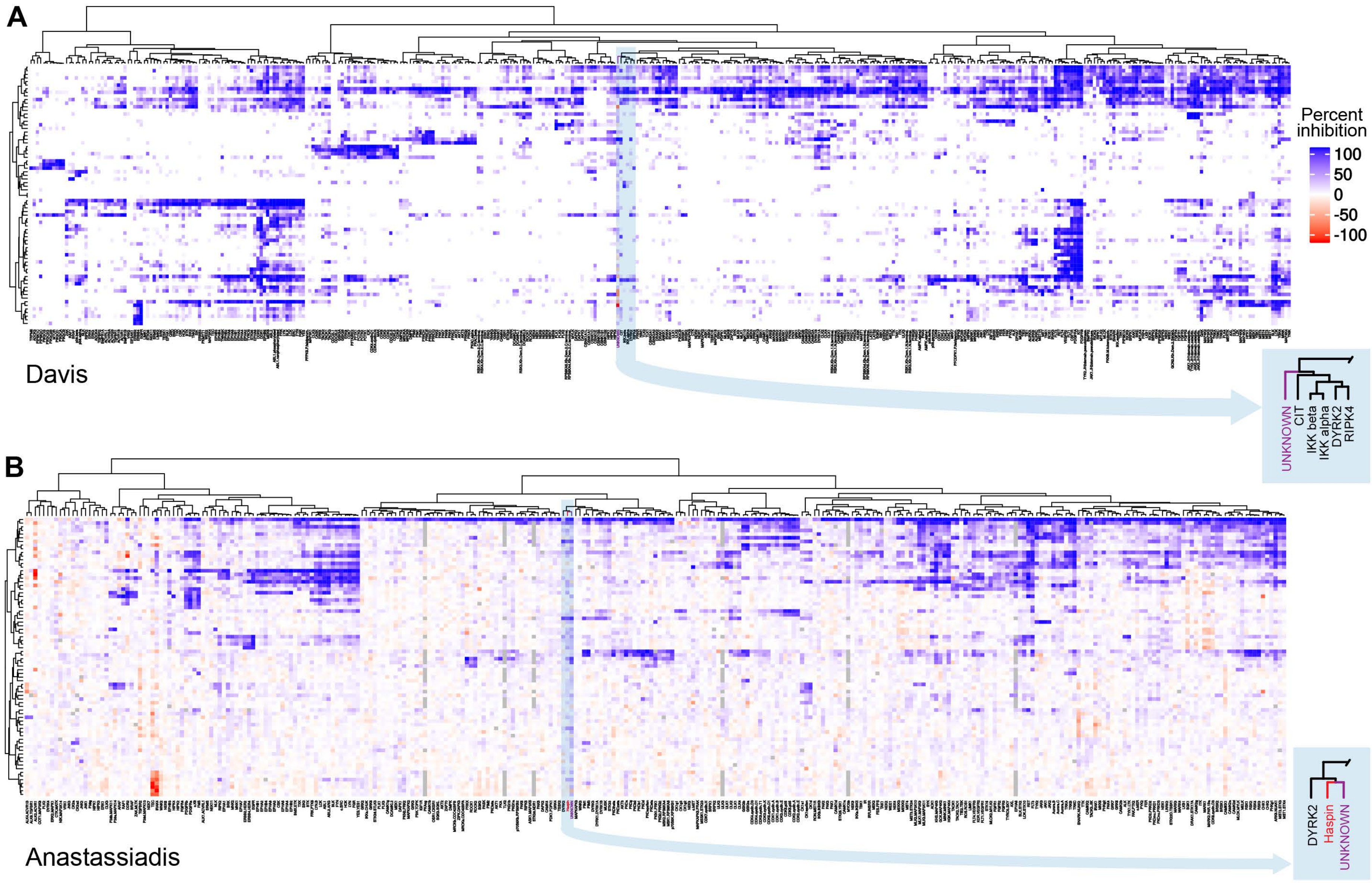

Supplementary Figure 22. Kinase dendrograms constructed from inhibition profiling data and KiPIK results for the H3T3ph kinase. Pale blue boxes show relevant portions of the dendrograms in greater detail. The "UNKNOWN" kinase is shown in purple, and the expected hit kinase Haspin/GSG2 in red. For details, see the legend to Supplementary Figure 21.

A. Profiling data from Davis *et al*.[6] which do not contain Haspin/GSG2.

B. Profiling data from Anastassiadis *et al*.[5] which do contain Haspin/GSG2.

A

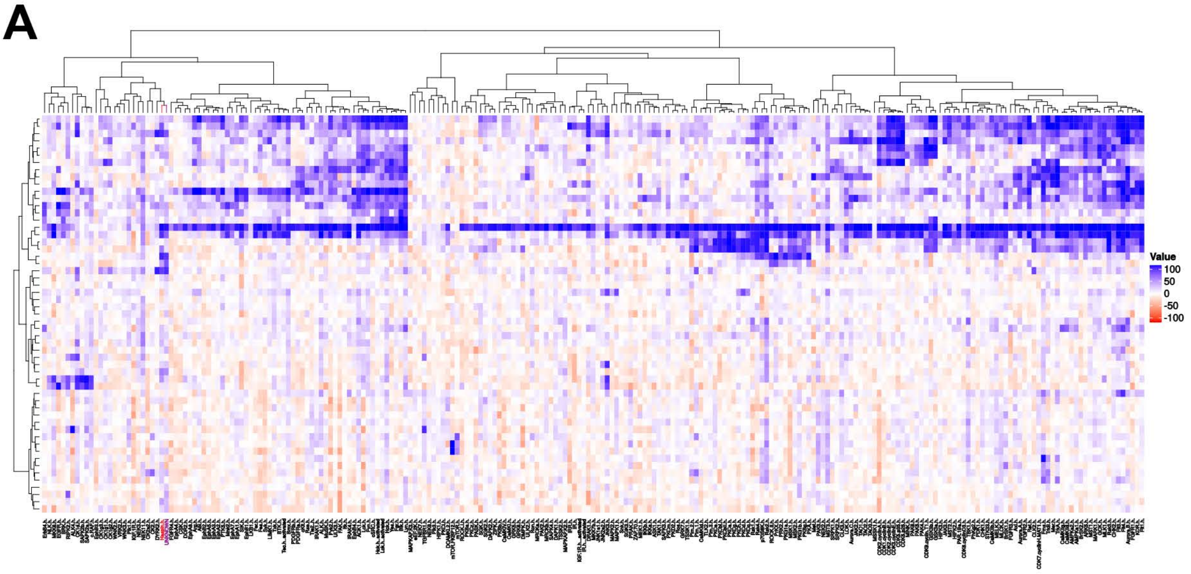

B

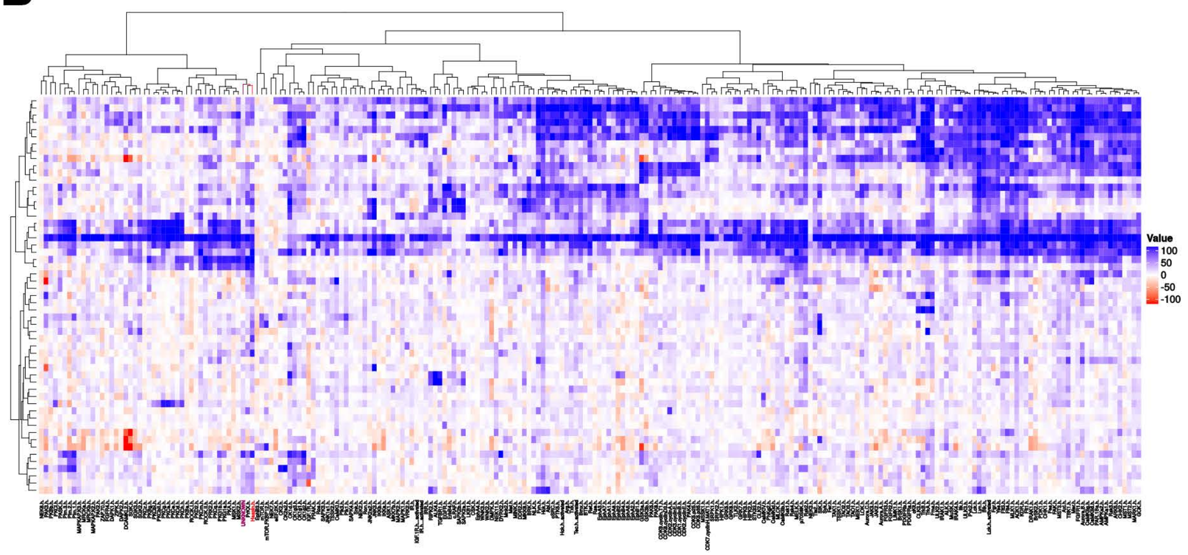

Supplementary Figure 23. Kinase dendrograms constructed from inhibition profiling data and KiPIK results for the H3T3ph kinase.

A. Gao 1 μM dataset. **B**. Gao 10 μM dataset.
The “UNKNOWN” kinase is shown in purple, and the expected hit kinase Haspin/GSG2 in red. See legend to Supplementary Figure 21 for details.

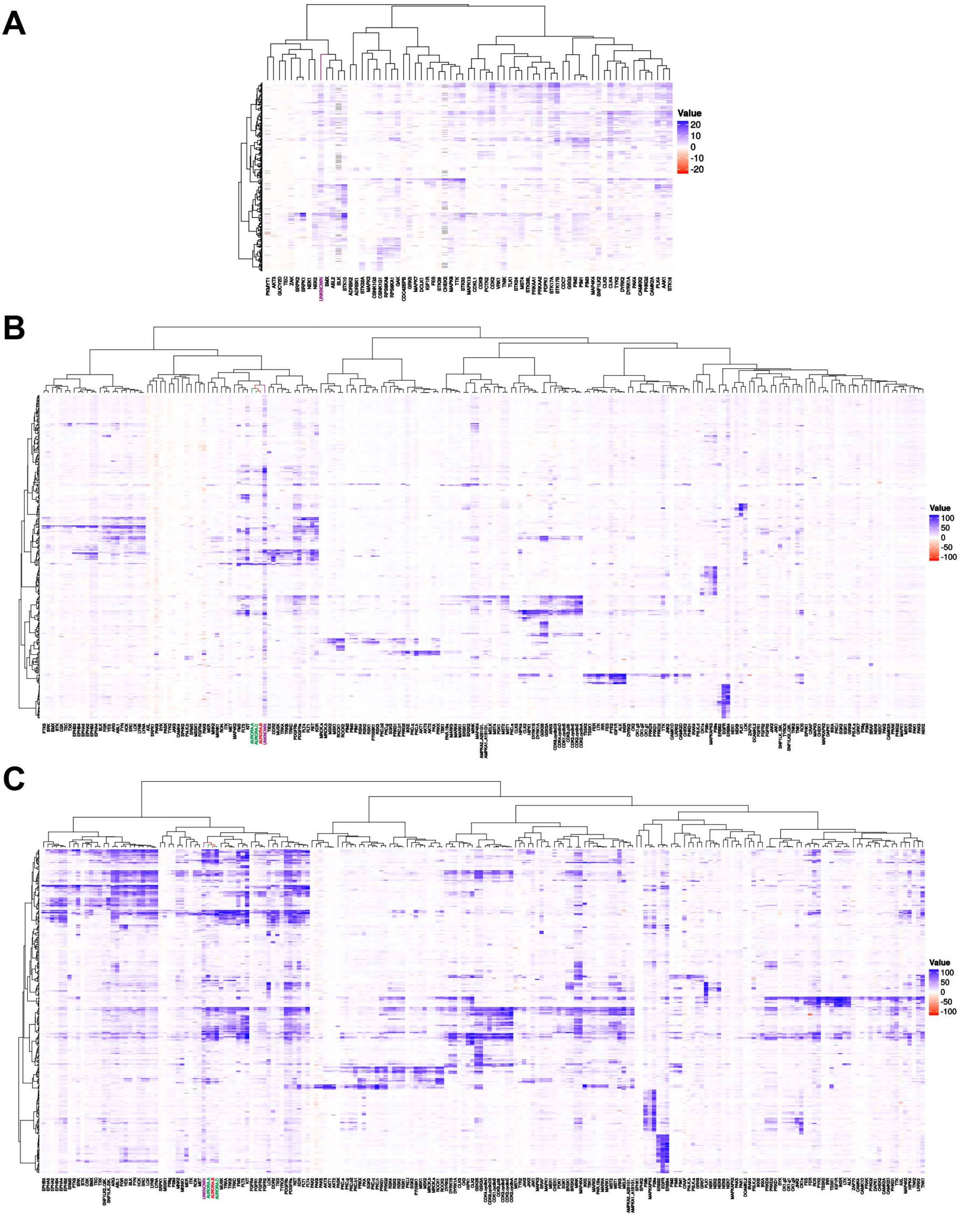

Supplementary Figure 24. Kinase dendrograms constructed from inhibition profiling data and KiPIK results for the H3S28ph kinase.

A. DSF dataset. Note that, for the DSF tree only, the KiPIK data are shown as %inhibition/10 so that they are on a similar scale. **B**. Nanosyn 0.1 μM dataset. **C**. Nanosyn 1 μM dataset.
The “UNKNOWN” kinase is shown in purple, the expected hit kinase Aurora B in red, and related kinases in green. See legend to Supplementary Figure 21 for details.

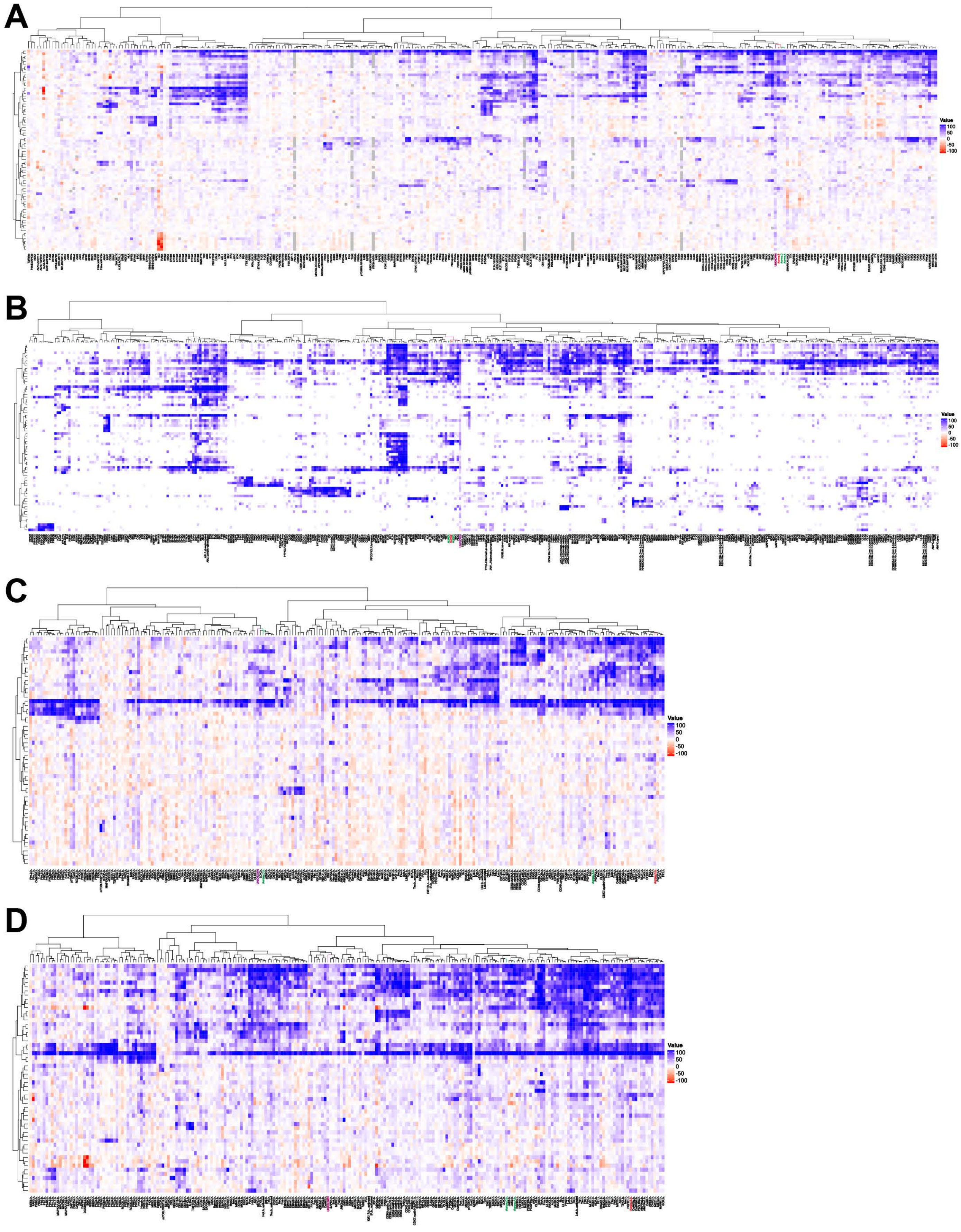

Supplementary Figure 25. Kinase dendrograms constructed from inhibition profiling data and KiPIK results for the H3S28ph kinase.

A. Anastassiadis dataset. **B**. Davis dataset. **C**. Gao 1 μM dataset. **D**. Gao 10 μM dataset.
The “UNKNOWN” kinase is shown in purple, the expected hit kinase Aurora B in red, and related kinases in green. See legend to Supplementary Figure 21 for details.

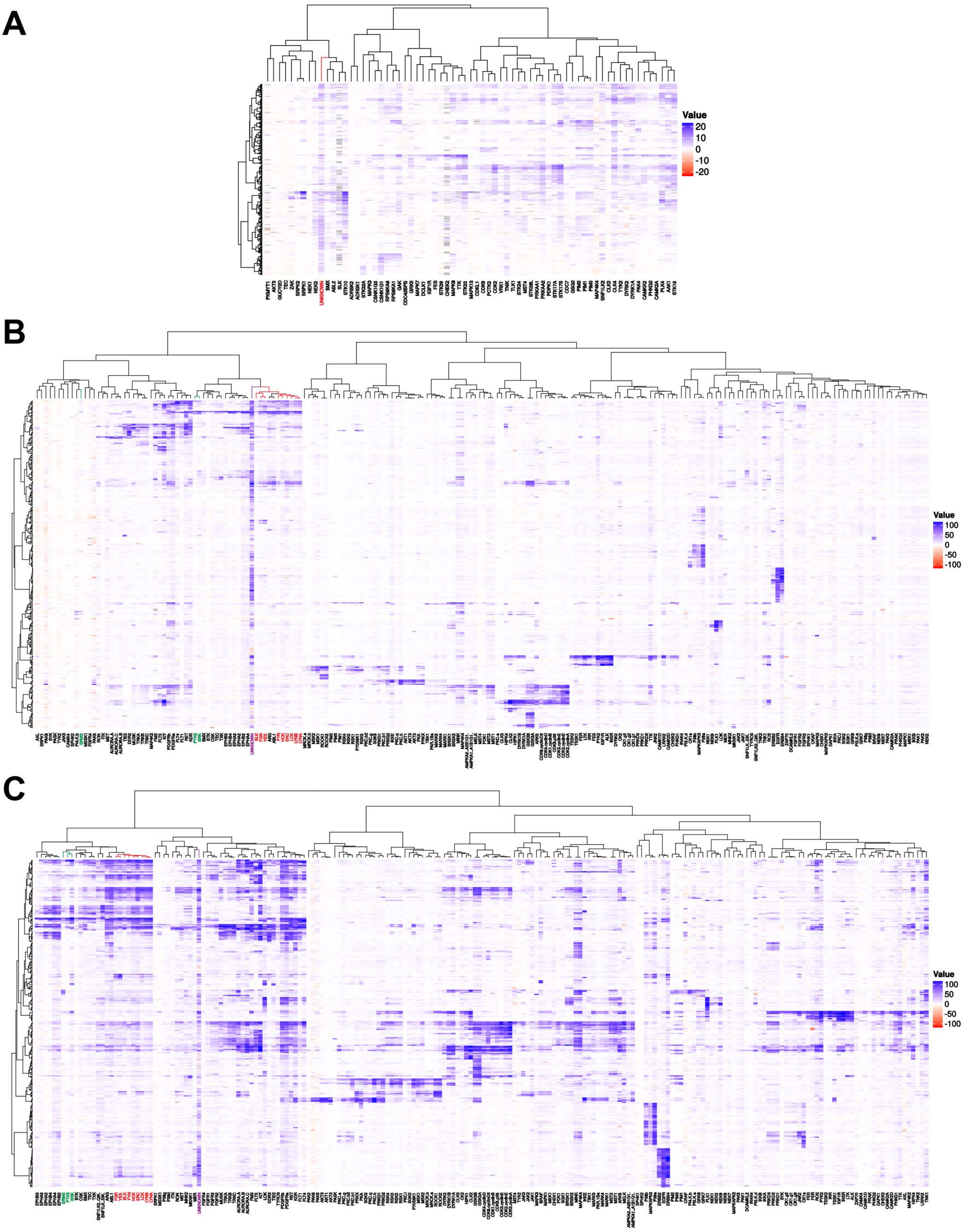

Supplementary Figure 26. Kinase dendrograms constructed from inhibition profiling data and KiPIK results for the Integrin β1A Y795ph kinase.

A. DSF dataset. Note that, for the DSF tree only, the KiPIK data are shown as %inhibition/10 so that they are on a similar scale. **B**. Nanosyn 0.1 μM dataset. **C**. Nanosyn 1 μM dataset.
The “UNKNOWN” kinase is shown in purple, the expected hit kinases of the SRC family in red, and related kinases in green. See legend to Supplementary Figure 21 for details.

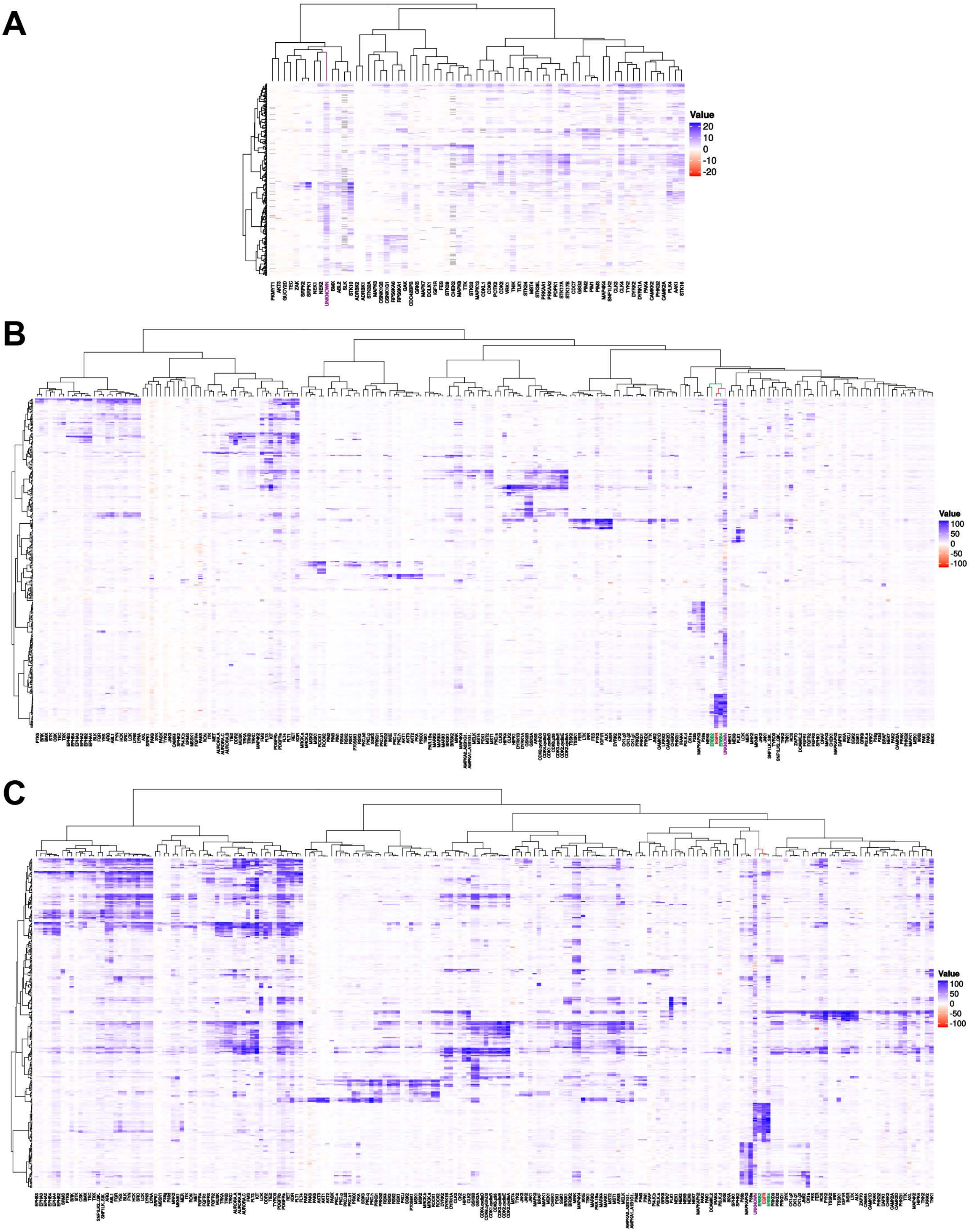

Supplementary Figure 27. Kinase dendrograms constructed from inhibition profiling data and KiPIK results for the EGFR Y1016ph kinase.

A. DSF dataset. Note that, for the DSF tree only, the KiPIK data are shown as %inhibition/10 so that they are on a similar scale. **B**. Nanosyn 0.1 μM dataset. **C**. Nanosyn 1 μM dataset.
The "UNKNOWN" kinase is shown in purple, the expected hit kinase EGFR in red, and related kinases in green. See legend to Supplementary Figure 21 for details.

A

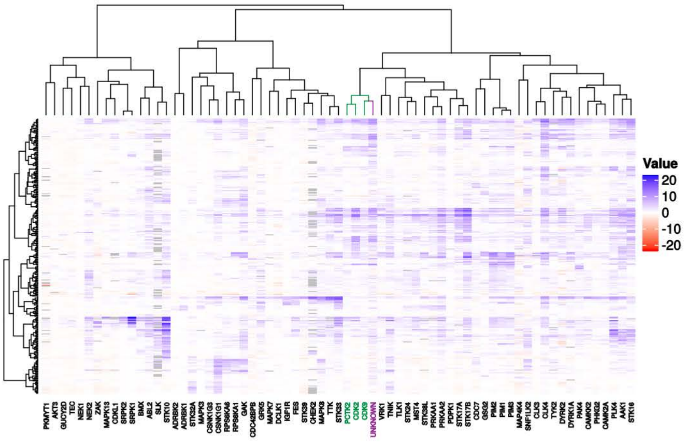

B

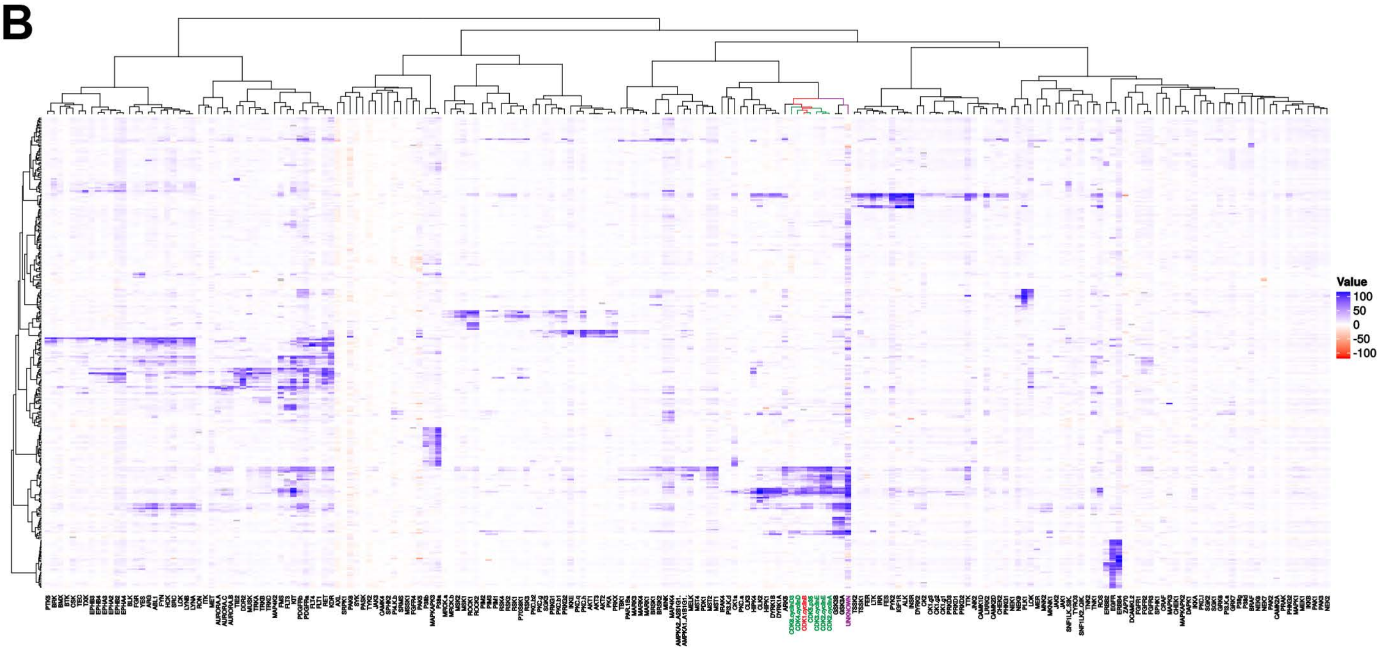

C

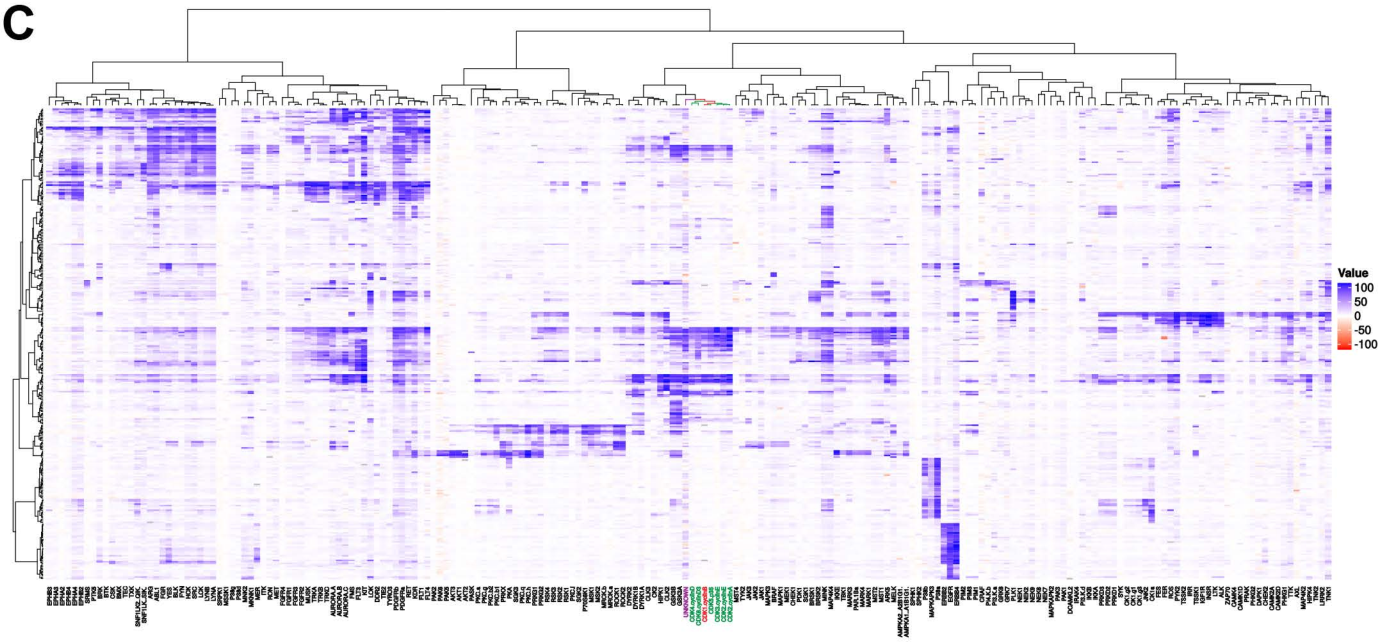

Supplementary Figure 28. Kinase dendrograms constructed from inhibition profiling data and KiPIK results for the INCENP S446ph kinase.

A. DSF dataset. Note that, for the DSF tree only, the KiPIK data are shown as %inhibition/10 so that they are on a similar scale. **B**. Nanosyn 0.1 μM dataset. **C**. Nanosyn 1 μM dataset.
The “UNKNOWN” kinase is shown in purple, the overall top hit kinase Cyclin B-Cdk1 in red, and related kinases in green. See legend to Supplementary Figure 21 for details.

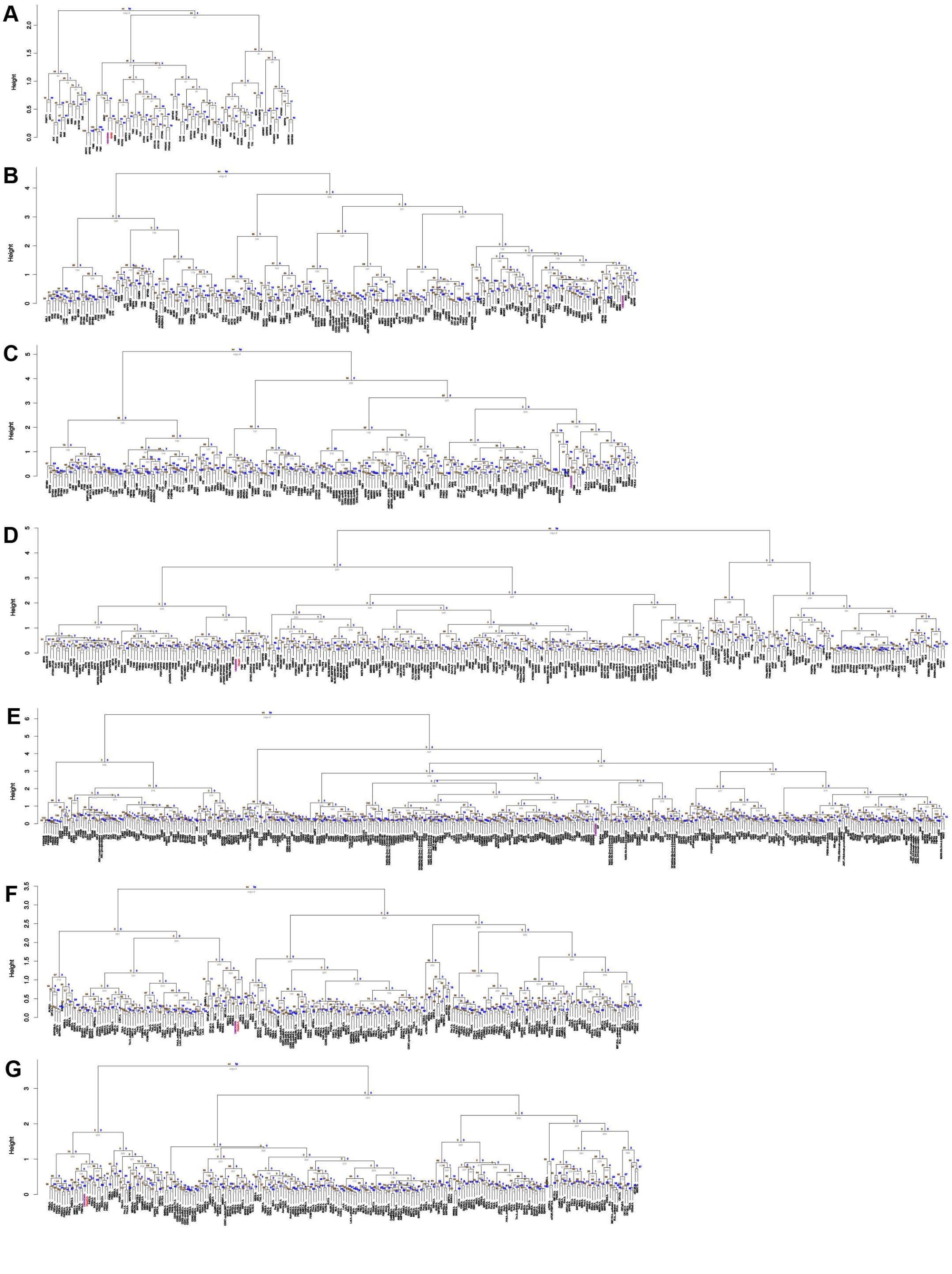

Supplementary Figure 29. Kinase dendrograms constructed from inhibition profiling data and KiPIK results for the H3T3ph kinase, with bootstrapping (10,000x).

A. DSF. **B**. Nanosyn 0.1 μM. **C**. Nanosyn 1 μM. **D**. Anastassiadis. **E**. Davis. **F**. Gao 1 μM. **G**. Gao 10 μM. The “UNKNOWN” kinase is shown in purple and the expected hit kinase Haspin/GSG2 in red. AU values are in brown, BP values in blue, and edge numbers in grey.

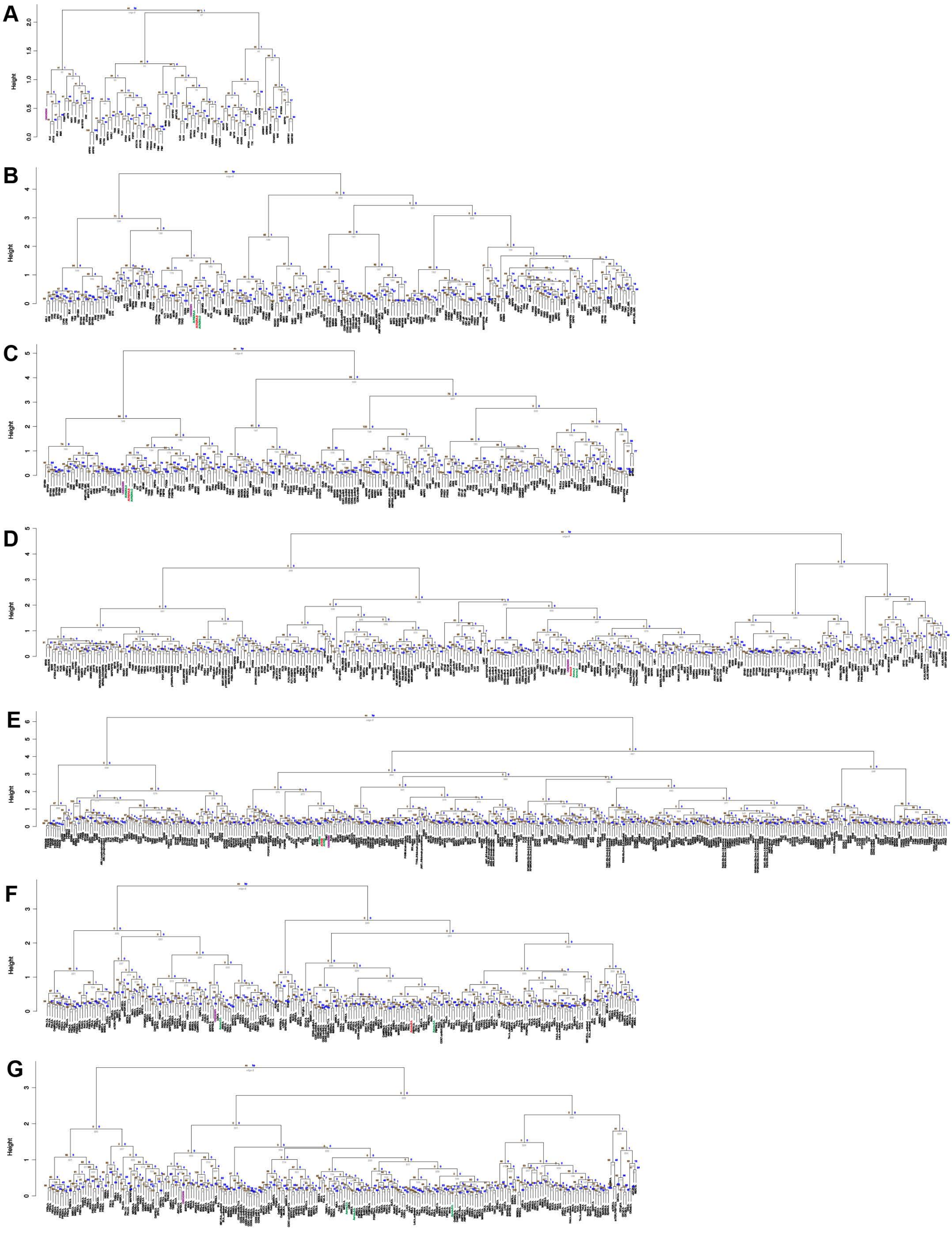

Supplementary Figure 30. Kinase dendrograms constructed from inhibition profiling data and KiPIK results for the H3S28ph kinase, with bootstrapping (10,000x).

A. DSF. **B**. Nanosyn 0.1 μM. **C**. Nanosyn 1 μM. **D**. Anastassiadis. **E**. Davis. **F**. Gao 1 μM. **G**. Gao 10 μM. The “UNKNOWN” kinase is shown in purple, the expected hit kinase Aurora B in red, and related kinases in green. AU values are in brown, BP values in blue, and edge numbers in grey.

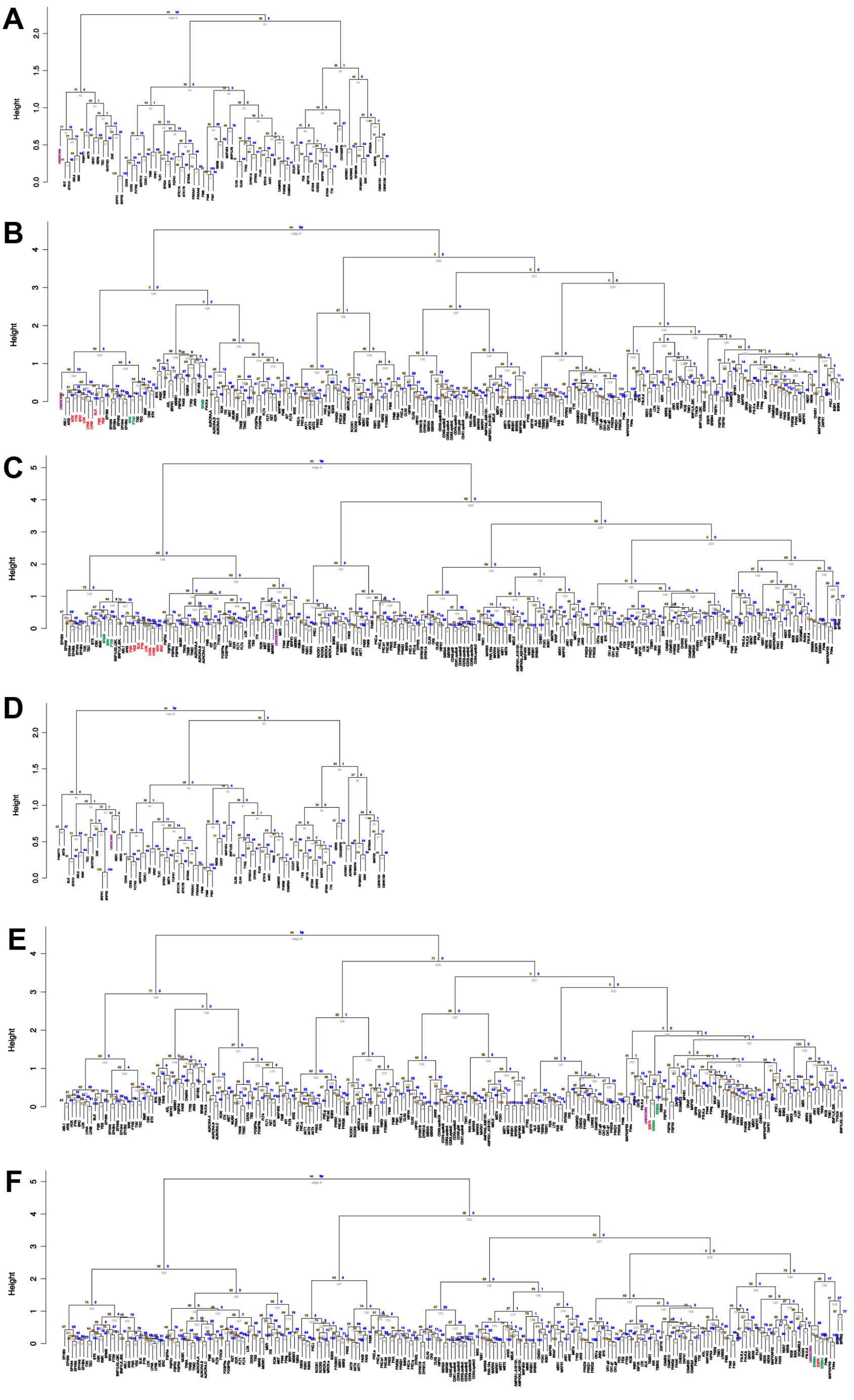

Supplementary Figure 31. Kinase dendrograms constructed from inhibition profiling data and KiPIK results for the Integrin β1A Y795ph and EGFR Y1016ph kinases, with bootstrapping (10,000x).

Integrin β1A Y795ph kinase: **A**. DSF. **B**. Nanosyn 0.1 µM. **C**. Nanosyn 1 µM.
EGFR Y1016ph kinase: **D**. DSF. **E**. Nanosyn 0.1 µM. **F**. Nanosyn 1 µM.
The “UNKNOWN” kinase is shown in purple, the expected hit kinases in red and related kinases in green. AU values are in brown, BP values in blue, and edge numbers in grey.

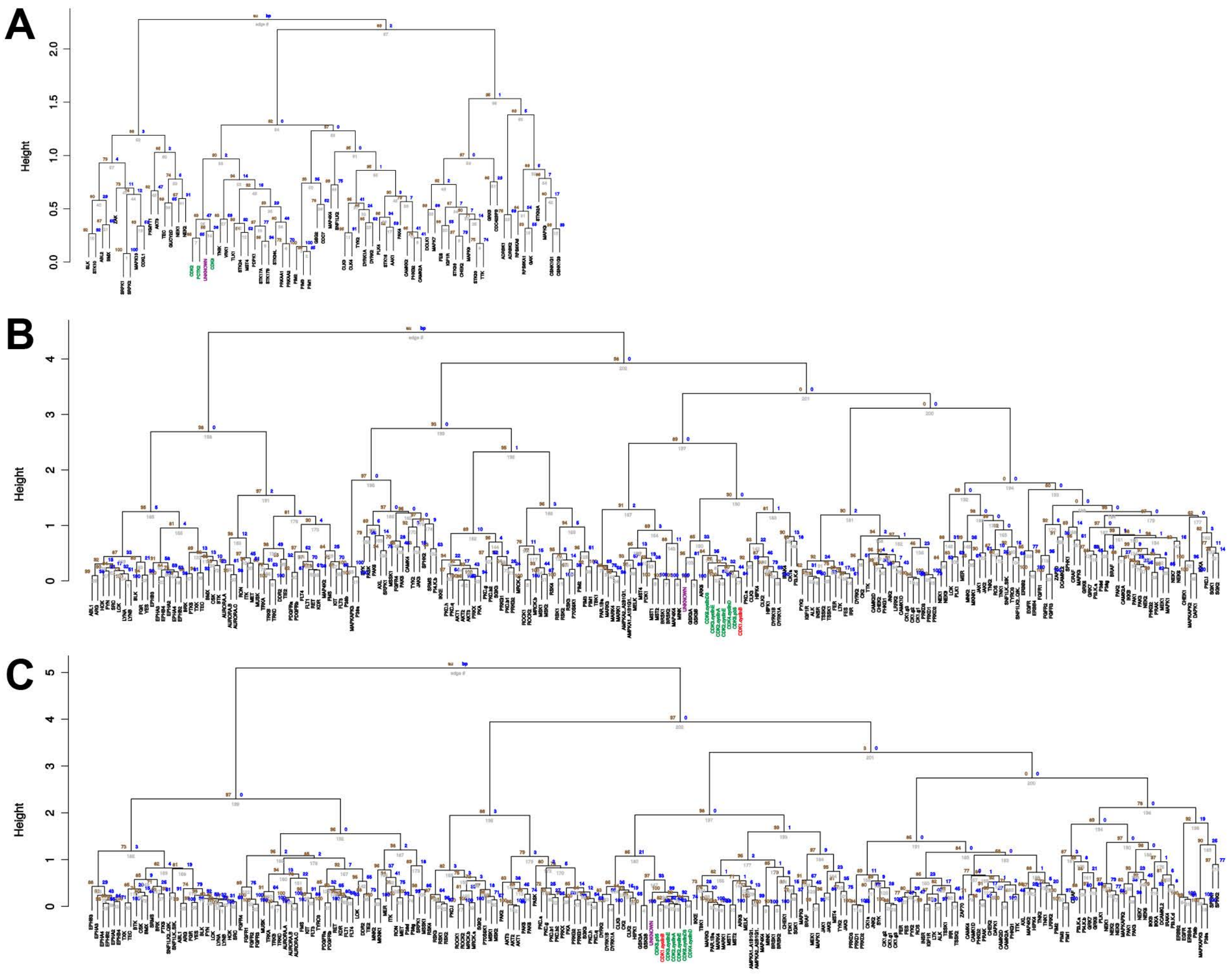

Supplementary Figure 32. Kinase dendrograms constructed from inhibition profiling data and KiPIK results for the INCENP S446ph kinase, with bootstrapping (10,000x).

A. DSF. **B**. Nanosyn 0.1 µM. **C**. Nanosyn 1 µM.

The “UNKNOWN” kinase is shown in purple, the overall top hit kinase Cyclin B-Cdk1 in red and related kinases in green. AU values are in brown, BP values in blue, and edge numbers in grey.

H3T3ph

	KinasePhos 2.0		NetPhos 3.1		NetPhorest 2.1		NetworKIN		PHOSIDA		PhosphoNET		ScanSite 4.0	
1	No prediction		CDC2/CDK1	0.463	GRK group	0.12	TTK	1.85	No prediction		DNAPK/PRKDC	51	No prediction	
2			GSK3	0.437	DAPK group	0.11	DAPK3	1			ATR	42		
3			CaM-II/CAMK2	0.436	PKC/PRKC group	0.1	PKCbeta/PRCKB	0.94			MEK7/MAP2K7	36		
4			PKG/PRKG	0.400	DMPK group	0.09	PKCalpha/PRKCA	0.6			MEK4/MAP2K4	25		
5			CKI/CSNK1	0.391	MAP2K group	0.07	PKCzeta/PRKCZ	0.4			HIPK4	21		
6			p38MAPK	0.361	CLK group	0.06	PKCdelta/PRKCD	0.33			ULK2	6		
7			DNAPK/PRKDC	0.341	DNAPK/PRKDC	0.06	GRK1	0.3			HIPK2	4		
8			CKII/CSNK2	0.293	TTK	0.05	GRK3	0.3			HIPK3	3		
9			CDK5	0.236	SLK group	0.04	GRK5	0.3			Trb3/TRIB3	2		
10			ATM	0.233	MST group	0.04	GRK4	0.3			HIPK1	2		

H3S28ph

	KinasePhos 2.0		NetPhos 3.1		NetPhorest 2.1		NetworKIN		PHOSIDA		PhosphoNET		ScanSite 4.0	
1	Aurora group	0.976391	PKA/PRKA	0.684	PKA/PRKA group	0.24	PKAalpha/PRKACA	1.38	PKA	N/A	MTOR/FRAP	409	PKC delta/PRKCD	0.466
2	ATM	0.923114	PKG/PRKG	0.574	PAK group	0.18	PAK1	1.36			PIM1	392	PKA/PRKACG	0.650
3	MAPK group	0.809572	PKC/PRKC	0.477	RCK/ROCK group	0.16	PKCbeta/PRCKB	1.24			PIM3	388	AURORA B	0.716
4	IKK/IKBKB	0.697333	GSK3	0.455	CLK group	0.14	PKCalpha/PRKCA	0.99			MSK1/RPS6KA5	387		
5	PKB/AKT group	0.666591	CaM-II/CAMK2	0.425	PIM1/3 group	0.13	PKAbeta/PRKACB	0.49			PKCb/PRKCB1	384		
6	CK1/CSNK1	0.549269	CKI/CSNK1	0.415	PKC/PRKC group	0.13	PKAgamma/PRKACG	0.49			PKCt/PRKCQ	378		
7	RSK/RPS6KA group	0.535955	CDC2/CDK1	0.406	DMPK group	0.12	PKCzeta/PRKCZ	0.46			PKCe/PRKCE	375		
8	STK4	0.51238	RSK/RPS6KA	0.379	ACTR2/2B TGFBR2 group	0.08	PKCdelta/PRKCD	0.36			PKCh/PRKCH	374		
9	CHK1/CHEK1	0.51238	DNAPK/PRKDC	0.357	PKD/PRKD group	0.07	PAK3	0.35			PKCd/PRKCD	372		
10	AKT1	0.5	ATM	0.319	PIM2	0.07	PAK2	0.35			PIM2	371		

Integrin β1A Y795ph

	KinasePhos 2.0		NetPhos 3.1		NetPhorest 2.1		NetworKIN		PHOSIDA		PhosphoNET		ScanSite 4.0	
1	No prediction		EGFR	0.456	Trk group	0.06	KDR	6.84	EGFR	N/A	ITK	391	No prediction	
2			INSR	0.403	Eph group	0.05	FLT1	0.37			TEC	382		
3			SRC	0.397	KDR FLT1 group	0.04	TRKA/NTRK1	0.22			ERBB2	377		
4					MAP2K group	0.04	TRKC/NTRK3	0.19			SRM/SRMS	375		
5							TRKB/NTRK2	0.00			BMX	375		
6											BTK	373		
7											EGFR	373		
8											ERBB4	370		
9											ALK	367		
10											RET	367		

EGFR Y1016ph

	KinasePhos 2.0		NetPhos 3.1		NetPhorest 2.1		NetworKIN		PHOSIDA		PhosphoNET		ScanSite 4.0	
1	EGFR	0.942926	No prediction		EGFR group	0.08	ERBB2	23.85	EGFR	N/A	ZAP70	573	EGFR	0.459
2	ZAP70	0.528317			Eph group	0.06	EGFR	14.31			SYK	572	FGR	0.470
3	PDGFR	0.527931									TEC	556	PDGFRB	0.562
4	JAK2	0.526844									ARG/ABL2	555	ABL1	0.673
5	IGF1R	0.523152									ALK	549		
6	BTK	0.522771									TXK	548		
7	FES	0.521712									AXL	548		
8	RET	0.518117									BLK	547		
9	EPH group	0.513706									KIT	547		
10	IR/INSR	0.507936									FGFR4	546		

INCENP S446ph

	KinasePhos 2.0		NetPhos 3.1		NetPhorest 2.1		NetworKIN		PHOSIDA		PhosphoNET		ScanSite 4.0	
1	ATM	0.886475	PKC/PRKC	0.692	PKC/PRKC group	0.39	CDK1	18.96	No prediction		PIM3	544	CDC2/CDK1	0.560
2	Aurora group	0.834161	GSK3	0.480	RCK/ROCK group	0.24					PIM1	538	PKC alpha/beta/gamma	0.569
3	CK1/CSNK1	0.797518	p38MAPK	0.436	CDK1/2/3/5 group	0.24					PIM2	536	CDK5	0.572
4	MAPK group	0.555368	CaM-II/CAMK2	0.419	PKB/AKT group	0.09					PKCb/PRKCB1	478	GSK3 group	0.602
5	RSK/RPS6KA group	0.540009	CKI/CSNK1	0.365							PKCt/PRKCQ	474	CDK1 motif 1	0.955
6	STK4	0.513056	DNAPK/PRKDC	0.347							MSK1/RPS6KA5	468	CDK1 motif 2	0.996
7	CHK1/CHEK1	0.513056	CDC2/CDK1	0.317							ASK1/MAP3K5	466		
8	AKT1	0.510758	RSK/RPS6KA	0.307							PKCa/PRKCA	462		
9	PKG/PRKG	0.5	ATM	0.286							PKCh/PRKCH	462		
10			CDK5	0.283							MAP3K15	459		

BCL9L S915ph

	KinasePhos 2.0		NetPhos 3.1		NetPhorest 2.1		NetworKIN		PHOSIDA		PhosphoNET		ScanSite 4.0	
1	ATM	0.955	PKA/PRKA	0.789	PKA/PRKA group	0.35	PAK1	1.43	PKA/PRKA	N/A	PIM1	721	PKA/PRKACG	0.328
2	PKB/AKT group	0.625	RSK/RPS6KA	0.554	DMPK group	0.20	PKAalpha/PRKACA	1.32	CAMK2	N/A	PIM3	711	AURORA B	0.516
3	PKA/PRKA	0.545	CaM-II/CAMK2	0.478	PAK group	0.18	PKAbeta/PRKACB	0.57	PKD/PRKD	N/A	PKG2/PRKG2	661	AURORA A	0.567
4	RSK/RPS6KA group	0.526	CDC2/CDK1	0.472	Pim1/3 group	0.14	PKAgamma/PRKACG	0.57	CHEK1/2	N/A	PIM2	641	AMPK/PRKAA1	0.604
5	STK4	0.513	PKG/PRKG	0.463	GRK group	0.12	PKCalpha/PRKCA	0.41			CHK2/CHEK2	612	AKT1	0.773
6	CHK1/CHEK1	0.513	GSK3	0.441	RCK/ROCK group	0.12	PAK3	0.35			MAPKAP3	611	CLK2	0.904
7	CK1/CSNK1	0.506	CKI/CSNK1	0.368	PKD/PRKD group	0.08	PAK2	0.35			MAPKAP2	609		
8	AKT1	0.500	DNAPK/PRKDC	0.344	CLK group	0.07	PAK4	0.35			MSK1/RPS6KA5	598		
9	PKG/PRKG	0.500	ATM	0.317	PKC/PRKC group	0.07	PAK6	0.35			PKG1/PRKG1	598		
10			PKB/AKT	0.247	AMPK/PRKAA group	0.07	PAK5	0.35			CAMK4	582		

Supplemental Figure 33

Supplementary Figure 33. Predicted kinases for specific phosphorylation sites using *in silico* approaches.

We used 7 different methods for which web-based tools are available to predict the most likely kinases for each of the phosphorylation sites used in KiPIK screening. Expected kinases are shown in red, and closely related kinases in green. None of the approaches was consistently able to predict the correct kinase. For example, although many methods identified EGFR as the likely kinase for EGFR Y1016ph, two of the methods failed to place this kinase in the top three (NetPhos 3.1, and PhosphoNET). None of the methods was able to place Haspin/GSG2 in the top ten places for H3T3ph. For each phosphosite, the number of predictions that placed the correct kinase(s) as the top candidate were: H3T3ph, 0/7; H3S28ph, 1/7; Integrin β1A Y795ph, 0/7; EGFR Y1016ph, 4/7; INCENP S446ph, 2/7; BCL9L S915ph, 4/7. This failure rate occurs even when the expected kinase is available to the prediction algorithms at high frequency: Haspin, 1/7; Aurora family, 6/7; Src family, 7/7; EGFR, 7/7; Cdk1, 7/7; PKA 7/7.

KinasePhos 2.0 (http://kinasephos2.mbc.nctu.edu.tw/index.html) uses sequence-based amino acid coupling pattern analysis and solvent accessibility in a SVM (support vector machine). Validated sites from the phospho.ELM and Swiss-Prot databases were used for training. Predictions can be made for 58 human protein kinases. The higher the score, the more likely the residue is phosphorylated[9].

NetPhos 3.1 (http://www.cbs.dtu.dk/services/NetPhos/) predicts phosphorylation sites using neural networks based on phosphorylation sites in the phospho.ELM database. Predictions are made for 17 kinases: ATM, CKI/CSNK1, CKII/CSNK2, CaM-II/CAMK2, DNAPK/PRKDC, EGFR, GSK3, INSR, PKA, PKB/AKT, PKC, PKG, RSK, SRC, CDC2/CDK1, CDK5 and p38MAPK. Output scores are in the range 0 to 1; scores above 0.5 indicate “positive predictions”[10].

NetPhorest 2.1 (http://www.netphorest.info/index.shtml) uses a collection of probabilistic classifiers based on position-specific scoring matrices (PSSM) or neural networks to classify phosphorylation sites according to the likely kinase responsible. It assumes that kinases that are on a similar branch of a sequence-based dendrogram have similar substrates and makes predictions for approximately 70 subgroups covering 222 kinases. Scores are from 0 to 1, with high scores being more confident predictions[11,12].

NetworKIN 3.0 (http://www.networkin.info/index.shtml) builds on NetPhorest by including contextual information on kinases and substrates from the STRING database, such as binding interactions or substrates that have other residues that are already known targets of a kinase. The theoretically neutral score is 1, and the higher the score for a given kinase, the higher the likelihood it is indeed the kinase. Kinome coverage is as for NetPhorest 2.1. Note that CDK1 is a strong “hit” for INCENP S446ph in part because the STRING database recognises that other INCENP residues are known targets for this kinase[12,13].

PHOSIDA (http://141.61.102.18/phosida/index.aspx) includes the simple Motif Matcher tool that searches for sequence matches with annotated kinase recognition patterns for 33 motifs covering 25 kinases. No score is provided[14].

PhosphoNET (http://www.phosphonet.ca) includes Kinase Predictor V2 that makes predictions based on determinants of specificity (DoS) within the primary amino acid sequences of the catalytic domains of 488 human protein kinases. The higher the score, the better the prospect that a kinase will phosphorylate a given site, with a maximum possible score of 1000 (ref. 15).

ScanSite 4.0 (https://scansite4.mit.edu/4.0/#home). Phosphorylation sites for particular kinases are predicted using PSSMs determined from oriented peptide library techniques. Thirty-three motifs covering 31 human kinases are included. Scores are on a scale of 0 to infinity, where 0 means a protein sequence perfectly matches the optimal binding pattern, and larger numbers indicate progressively poorer matches to the optimal consensus sequence[16,17].

Supplementary References

1 Eid, S., Turk, S., Volkamer, A., Rippmann, F. & Fulle, S. KinMap: a web-based tool for interactive navigation through human kinome data. *BMC Bioinformatics* **18**, 16 (2017).

2 Elkins, J. M. *et al.* Comprehensive characterization of the Published Kinase Inhibitor Set. *Nat Biotechnol* **34**, 95-103 (2016).

3 Hummer, S. & Mayer, T. U. Cdk1 Negatively Regulates Midzone Localization of the Mitotic Kinesin Mklp2 and the Chromosomal Passenger Complex. *Curr Biol* **19**, 607-612 (2009).

4 Dai, J., Sultan, S., Taylor, S. S. & Higgins, J. M. G. The kinase haspin is required for mitotic histone H3 Thr 3 phosphorylation and normal metaphase chromosome alignment. *Genes Dev* **19**, 472-488 (2005).

5 Anastassiadis, T., Deacon, S. W., Devarajan, K., Ma, H. & Peterson, J. R. Comprehensive assay of kinase catalytic activity reveals features of kinase inhibitor selectivity. *Nat Biotechnol* **29**, 1039-1045 (2011).

6 Davis, M. I. *et al.* Comprehensive analysis of kinase inhibitor selectivity. *Nat Biotechnol* **29**, 1046-1051 (2011).

7 Gao, Y. *et al.* A broad activity screen in support of a chemogenomic map for kinase signalling research and drug discovery. *Biochem J* **451**, 313-328 (2013).

8 Drewry, D. H. *et al.* Progress towards a public chemogenomic set for protein kinases and a call for contributions. *PLoS One* **12**, e0181585 (2017).

9 Wong, Y. H. *et al.* KinasePhos 2.0: a web server for identifying protein kinase-specific phosphorylation sites based on sequences and coupling patterns. *Nucleic Acids Res* **35**, W588-594 (2007).

10 Blom, N., Sicheritz-Ponten, T., Gupta, R., Gammeltoft, S. & Brunak, S. Prediction of post-translational glycosylation and phosphorylation of proteins from the amino acid sequence. *Proteomics* **4**, 1633-1649 (2004).

11 Miller, M. L. *et al.* Linear motif atlas for phosphorylation-dependent signaling. *Sci Signal* **1**, ra2 (2008).

12 Horn, H. *et al.* KinomeXplorer: an integrated platform for kinome biology studies. *Nat Methods* **11**, 603-604 (2014).

13 Linding, R. *et al.* Systematic discovery of in vivo phosphorylation networks. *Cell* **129**, 1415-1426 (2007).

14 Gnad, F., Gunawardena, J. & Mann, M. PHOSIDA 2011: the posttranslational modification database. *Nucleic Acids Res* **39**, D253-260 (2011).

15 Safaei, J., Manuch, J., Gupta, A., Stacho, L. & Pelech, S. Prediction of 492 human protein kinase substrate specificities. *Proteome Sci* **9 Suppl 1**, S6 (2011).

16 Yaffe, M. B. *et al.* A motif-based profile scanning approach for genome-wide prediction of signaling pathways. *Nat Biotechnol* **19**, 348-353 (2001).

17 Obenauer, J. C., Cantley, L. C. & Yaffe, M. B. Scansite 2.0: Proteome-wide prediction of cell signaling interactions using short sequence motifs. *Nucleic Acids Res* **31**, 3635-3641 (2003).
