# Supplementary material for: Kinase inhibition profiles as a tool to identify kinases for specific phosphorylation sites
Source: Nat Commun. 2020 Apr 3;11:1684. doi: 10.1038/s41467-020-15428-0 (PMC7125195; doi:10.1038/s41467-020-15428-0)
Supplement: Supplementary file 3 — Description of Additional Supplementary Files [file 41467_2020_15428_MOESM3_ESM.docx]

**Description of Additional Supplementary Files**

File name: Supplementary Data 1

Description: Kinases profiled.

The table lists all the kinases profiled in the Nanosyn, DSF, PKIS2, Davis, Anastassiadis and Gao datasets used in this study. The kinase names used are those in the original published profiling studies. In addition, the HGNC symbols for each kinase are provided. The total numbers of kinases profiled are shown at the bottom.

File name: Supplementary Data 2

Description: PKIS1 library.

PKIS1 was supplied by GlaxoSmithKline LLC and the Structural Genomics Consortium under an open access Material Transfer and Trust Agreement: <http://www.sgc-unc.org>. Note that the inhibitor highlighted in red was profiled in the DSF but not Nanosyn datasets.

File name: Supplementary Data 3

Description: Custom library.

Details of the 128 kinase inhibitors constituting the custom library.

File name: Supplementary Data 4

Description: PKIS2 library.

PKIS2 was supplied by GlaxoSmithKline LLC and the Structural Genomics Consortium under an open access Material Transfer and Trust Agreement: <http://www.sgc-unc.org>. Note that inhibitors highlighted in red were not used in the "PKIS2 Repeat 1" experiment.
