# Supplementary material for: Kinase inhibition profiles as a tool to identify kinases for specific phosphorylation sites
Source: Nat Commun. 2020 Apr 3;11:1684. doi: 10.1038/s41467-020-15428-0 (PMC7125195; doi:10.1038/s41467-020-15428-0)
Supplement: Supplementary file 8 — Reporting Summary [file 41467_2020_15428_MOESM8_ESM.pdf]

nature research

Corresponding author(s): Higgins, J.M.G.

Last updated by author(s): 01 Mar 2020

Reporting Summary

Nature Research wishes to improve the reproducibility of the work that we publish. This form provides structure for consistency and transparency in reporting. For further information on Nature Research policies, see Authors & Referees and the Editorial Policy Checklist .

Statistics

For all statistical analyses, confirm that the following items are present in the figure legend, table legend, main text, or Methods section.

n/a	Confirmed	
☐	☒	The exact sample size (*n*) for each experimental group/condition, given as a discrete number and unit of measurement
☐	☒	A statement on whether measurements were taken from distinct samples or whether the same sample was measured repeatedly
☐	☒	The statistical test(s) used AND whether they are one- or two-sided<br>*Only common tests should be described solely by name; describe more complex techniques in the Methods section.*
☒	☐	A description of all covariates tested
☒	☐	A description of any assumptions or corrections, such as tests of normality and adjustment for multiple comparisons
☐	☒	A full description of the statistical parameters including central tendency (e.g. means) or other basic estimates (e.g. regression coefficient) AND variation (e.g. standard deviation) or associated estimates of uncertainty (e.g. confidence intervals)
☐	☒	For null hypothesis testing, the test statistic (e.g. *F*, *t*, *r*) with confidence intervals, effect sizes, degrees of freedom and *P* value noted<br>*Give P values as exact values whenever suitable.*
☒	☐	For Bayesian analysis, information on the choice of priors and Markov chain Monte Carlo settings
☒	☐	For hierarchical and complex designs, identification of the appropriate level for tests and full reporting of outcomes
☐	☒	Estimates of effect sizes (e.g. Cohen's *d*, Pearson's *r*), indicating how they were calculated

Our web collection on statistics for biologists contains articles on many of the points above.

Software and code

Policy information about availability of computer code

Data collection	For RNAi screens, we used the High Content Analysis software in Nikon Elements with JOBS 4.12. For immunofluorescence microscopy we used ZEN 2.3 (Zeiss).
Data analysis	For analyzing KiPIK data we used Microsoft Excel 16.16 and GraphPad Prism 7.0b. For hierarchical clustering of inhibition fingerprints, we used hclust and heatmap in the ComplexHeatmap v1.10.2 package implemented in R version 3.4.0 (2017-04-21). For bootstrapping, we used hclust within pvclust package 4. For inhibitor downsampling we used the cor function in R. R source code can be found at https://github.com/CnrLwlss/Watson_2020 and the VBA Shuffler code is provided in the Source Data file.

For manuscripts utilizing custom algorithms or software that are central to the research but not yet described in published literature, software must be made available to editors/reviewers. We strongly encourage code deposition in a community repository (e.g. GitHub). See the Nature Research guidelines for submitting code & software for further information.

Data

Policy information about availability of data

All manuscripts must include a data availability statement. This statement should provide the following information, where applicable:

- Accession codes, unique identifiers, or web links for publicly available datasets
- A list of figures that have associated raw data
- A description of any restrictions on data availability

All relevant data are available from the authors. The source data underlying Figs 2, 3, 4A-C, 5 to 7 and Supplementary Figs 2 to 8 and 11 to 20 are provided as a Source Data file. Data for Supplementary Figs 9, 10, and 21 to 32 can also be found at https://github.com/CnrLwlss/Watson_2020.

Field-specific reporting

Please select the one below that is the best fit for your research. If you are not sure, read the appropriate sections before making your selection.

- [x] Life sciences
- [ ] Behavioural & social sciences
- [ ] Ecological, evolutionary & environmental sciences

For a reference copy of the document with all sections, see nature.com/documents/nr-reporting-summary-flat.pdf

Life sciences study design

All studies must disclose on these points even when the disclosure is negative.

Sample size	Sample size for KiPIK screening (n=2), RNAi screening (n=4), acute inhibitor treatment of cells (n=3), and in vitro peptide phosphorylation (n=6) was determined by the practicality of plate handling, cost of reagents etc. These sizes were sufficient as determined by the calculation of z-scores and standard scores and reproducing known kinase-substrate pairs.
Data exclusions	The siRNA screening panel we used focused on protein kinases but additional genes were present due to the way reagents were pre-arranged on plates. As pre-determined, only the results of RNAi directed at protein kinases were used in the main figure. The full data set is provided in Source Data. When conducting siRNA experiments to confirm BCL9L S915ph kinases, experiments where the positive control depletion of BCL9L itself was ineffective were excluded. Otherwise, no data exclusions were made.
Replication	KiPIK screens for H3T3ph and H3S28ph were repeated with alternative inhibitor libraries and alternative profiling datasets, and computational downsampling of inhibitor number was used to determine if the findings were reproducible using fewer inhibitors, which was found to be the case within limits (see paper). In addition, the BCL9L S915 KiPIK screen was carried out twice with essentially the same inhibitor library to test reproducibility, with similar results, as shown in the paper. The RNAi screen was repeated four times on separate plates, and results were combined to calculate standard scores and p values as shown in Figure 3 and Source Data.
Randomization	Randomization was not relevant because experiments involved treatments of cells derived from a single cell culture.
Blinding	All quantitative analysis was done using automated systems, and thus was effectively blinded.

Reporting for specific materials, systems and methods

We require information from authors about some types of materials, experimental systems and methods used in many studies. Here, indicate whether each material, system or method listed is relevant to your study. If you are not sure if a list item applies to your research, read the appropriate section before selecting a response.

Materials & experimental systems

n/a	Involved in the study	
[ ]	[x]	Antibodies
[ ]	[x]	Eukaryotic cell lines
[x]	[ ]	Palaeontology
[x]	[ ]	Animals and other organisms
[x]	[ ]	Human research participants
[x]	[ ]	Clinical data

Methods

n/a	Involved in the study	
[x]	[ ]	ChIP-seq
[x]	[ ]	Flow cytometry
[x]	[ ]	MRI-based neuroimaging

Antibodies

Antibodies used

Rabbit polyclonal antibodies:
H3T3ph (B8634; Jonathan Higgins)
H2BS6ph (Dr Lienhard Schmitz, Justus-Liebig-University, Germany)
INCENP (P240, Cell Signaling Technology #2807, Lot 1)
INCENP-S446ph (A771, Dr Jan-Michael Peters, IMP, Vienna)
INCENP-TSSph (Dr Michael Lampson, University of Pennsylvania)
BCL9L S915ph (Cell Signaling Technology #13325, Lot 1)
Neurogranin S36ph (Merck-Millipore ABN426, Lot Q2441540)
Gamma-tubulin (AK-15, Sigma, T3320, Lot 053M4774)

Rabbit monoclonal antibodies:
S-pT-P motif antibody (D73F6; Cell Signaling Technology #5243, Lot 1)
Cyclin B1 (D5C10; Cell Signaling Technology #12231, Lot 7)
Vinculin (E1E9V, Cell Signaling Technology #13901, Lot 5)

	Mouse monoclonal antibodies:<br>H3T3ph (16B2; Hiroshi Kimura)<br>H3S28ph (CMA315; Hiroshi Kimura)<br>Phospho-tyrosine (P-Tyr-100; Cell Signaling Technology #9411, Lot 25)<br><br>Sheep polyclonal antibodies:<br>Aurora B (SAB.1, Dr Stephen Taylor, University of Manchester)<br>BCL9L (R&D Systems AF4967, Lot CAYR0116081)<br><br>Donkey polyclonal antibodies:<br>anti-sheep IgG-HRP (Thermofisher A16041, Lot 58-120-041018)<br>anti-mouse Alexa Fluor 488 (ThermoFisher A-21202, Lot 1423052)<br>anti-rabbit Alexa Fluor 594 (ThermoFisher A-21207, Lot 1744751)<br>anti-sheep Alexa 488 Fluor (ThermoFisher A-11015, Lot 1807723)<br><br>Horse polyclonal antibodies:<br>anti-mouse IgG-HRP (Cell Signaling Technology #7076, Lot 33)<br><br>Goat polyclonal antibodies:<br>anti-rabbit IgG-HRP (Cell Signaling Technology #7074, Lot 28)
Validation	H3T3ph (B8634; ref 47); H3T3ph (16B2; ref 85); H3S28ph (CMA315; ref 83); H2BS6ph (ref 86); INCENP-S446ph (ref 57 and this study where an INCENP peptide containing S446 is detected only after phosphorylation in vitro); INCENP-TSSph (ref 82); INCENP P240, Cyclin B1, S-pT-P, and P-Tyr-100 (see CST website); BCL9L S915ph (see CST website, and this study where a BCL9L peptide containing S915 is detected only after phosphorylation in vitro, and reactivity is lost after BCL9L RNAi in cells); Neurogranin S36ph (see Merck website, and this study where a Neurogranin peptide containing S36 is detected only after phosphorylation in vitro); BCL9L (see R&D website and Mieszczanek et al. Nat Commun. 2019 10:724); Aurora B (SAB.1; ref 84); Gamma-tubulin (see Sigma website and size appropriate on blot); Vinculin (see CST website and size appropriate on blot).

Eukaryotic cell lines

Policy information about cell lines

Cell line source(s)	HeLa S3 cells were obtained from the ATCC (ATCC CCL 2.2). A431 cells were obtained from the laboratory of Dr Michael Brenner, Brigham and Women's Hospital, Boston, USA, and originally from the ATCC (ATCC CCR-1555).
Authentication	HeLa S3 and A431 cells were authenticated by the ATCC and not subsequently retested for authenticity.
Mycoplasma contamination	Cells were free from mycoplasma as determined by fluorescence microscopy with DNA dyes and periodic PCR-based assay.
Commonly misidentified lines<br>(See ICLAC register)	Commonly misidentified cell lines were not used in this study.
